# Supplementary material for: Lassa virus persistence with high viral titers following experimental infection in its natural reservoir host, Mastomys natalensis
Source: Nat Commun. 2024 Oct 29;15:9319. doi: 10.1038/s41467-024-53616-4 (PMC11522386; doi:10.1038/s41467-024-53616-4)
Supplement: Supplementary file 1 — Supplementary Information [file 41467_2024_53616_MOESM1_ESM.pdf]

Lassa virus persistence with high viral titers following experimental infection in its natural reservoir host, *Mastomys natalensis*

Supplementary Methods

Animals and monitoring. The breeding colony at the BNITM facilities is kept outbred, with 1:1 pairing. Health monitoring of the colony is done according to FELASA guidelines using sentinel animals (mouse strain RjOrl:SWISS, Janvier Labs, Le Genest-Saint-Isle, France) kept under the same conditions that are exposed to bedding, droppings, and food from *M. natalensis* cages. Animals from the breeding colony on occasion test positive for *Helicobacter sp*.. Otherwise, the breeding colony is tested free of infectious agents outlined in the official FELASA recommendations[1]. Animals were provided with cage enrichment, including houses, swings and running wheels. Regular food was on occasion supplemented with nuts (peanuts, walnuts, hazelnuts), sunflower seeds or dried millet. The permanent marking of Mastomys younger than 2 weeks is challenging, moreover, reduction of a litter could result in the loss of the whole litter due to stress-based parental aggression, thus, whole litters were used for all experiments. Juvenile Mastomys were weaned at the age of three weeks. The sex of animals was determined at the time of weaning. Males and females were distributed equally for sample collection (terminal sampling) based on initial litter composition. Animals younger than three weeks with unknown sex were chosen at random for terminal sampling. The number of animals euthanized for terminal sampling varied based on initial litter size and composition, however, for each experimental group at least two individuals were sacrificed per sampling period. To ensure the safety of experimenters and animals, additional safety nets and cut-resistant gloves (ActivArmr®, Carl Roth, Karlsruhe, Germany) were used while handling *Mastomys natalensis* during the experiments. Animals were trained to seek out and enter boxes (Anesthesia induction chamber, UNO Life Science Solutions, Zevenaar, Netherlands), for taking body weight or changing cages therefore no anesthesia was required for the day-to-day handling.

Virus strain. Experimental virus stocks are regularly checked for mycoplasma contamination by PCR and for the absence of mutations by Next Generation Sequencing.

Inoculation. Neonates showed strong reactions to anesthesia. However, animals younger than two weeks display a strong pinch-induced behavioral inhibition once grasped in the scruff of the neck and thus were inoculated without anesthesia to avoid complications. Individuals aged two weeks or older were anesthetized with isoflurane for the procedure. Following anesthesia animals were closely monitored until they fully regained consciousness. Inoculated neonates and juveniles were kept separated from their parents for at least 20 min, before returning the adult animals into the cage to prevent aggression from parents towards offspring. Two whole litters per experiment group were inoculated. In order to reduce the number of animals needed the inoculation experiments were designed based on the assumption that two breeding pairs per experimental group are representative of the different genetic backgrounds within our outbred colony. Naïve females inoculated during gestation gave birth to one litter each. No randomization or blinding of experimental groups was performed.

Transmission experiments. All animals used for breeding were at least 12 weeks old. Persistently infected females (n = 4) used in the breeding experiments originated from either day 6 inoculation (n = 2) or were borne to a female inoculated during pregnancy (n = 2). A total of four breeding pairs was used for the initial round. Two breeding pairs were used for each subsequent generation. Based on breeding performance and litter size across the whole group, each female gave birth to 2–4 litters. All persistently infected males used for breeding were born to infected mothers. Infected males were used for multiple breeding rounds and always paired with a naïve female for each round. Depending on breeding performance and litter size each male fathered 2–3 litters.

Sampling procedures. Amniotic fluid was collected by puncturing the amniotic sac after removing the conceptus from the uterus of pregnant females (<14 days into gestation) following euthanasia. Epididymal plasma (cell-free content of the epididymis) was gained by

dissecting the caudal part of the epididymis in 1 mL PBS, followed by incubation for 5 min at RT. Spermatozoa and cell debris were removed via centrifugation at 500x g for 5 min. The supernatant was used for further analysis.

***In vitro* infectivity assays.** The tested body fluids originate from animals inoculated at day 6 or individuals borne to persistently infected females. Samples were chosen based on virus titer (≥10^6 copies/mL) and sample availability.

Neutralization capacity of LASV-specific antibodies. The tested plasma samples were obtained from animals that were inoculated with LASV strain Ba366 (n = 37), offspring borne to infected mothers (n = 28), or adult animals exposed to infected individuals (n = 15). Anti-NP-specific IgG-positive samples (determined by ELISA) from viremic and non-viremic animals were selected based on sample availability covering a time span of 14–240 days post initial exposure.

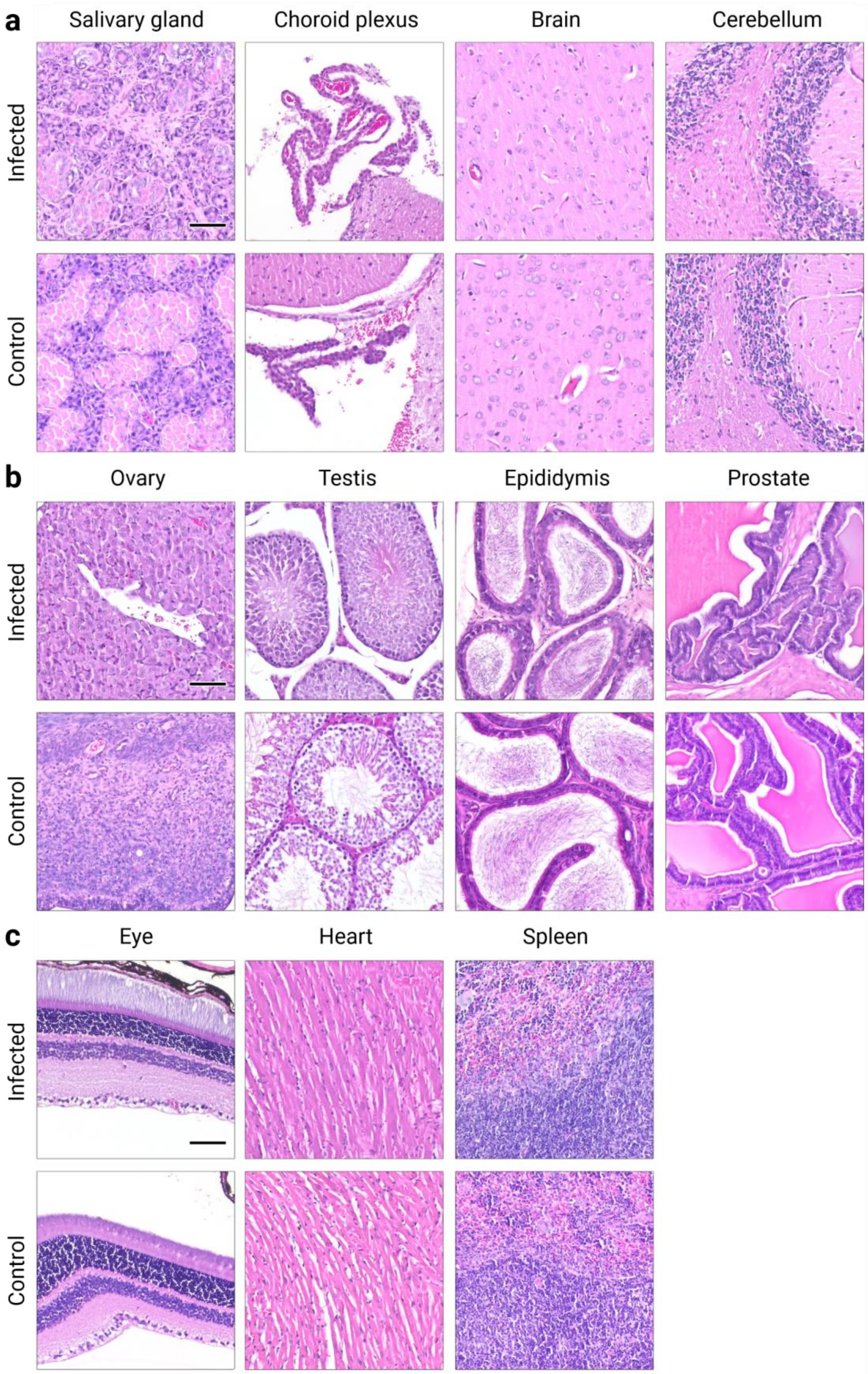

Supplementary Fig. 1. Histological analysis of organs following inoculation with LASV. Organs of female and male Mastomys inoculated s.c. with 1,000 FFU of LASV Ba366 on day 6–7 (n = 5) were compared to uninfected control (n = 2) animals. Infected animals (≥120 days post-inoculation) displayed no signs of immune infiltration, cell loss, or degeneration. Representative images of H&E-stained tissue sections are shown (from left to right): salivary gland, choroid plexus, brain, cerebellum (**a**); ovary, testis, epididymis, prostate (**b**); eye, heart, and spleen (**c**). Black scale bar equals 50 µm.

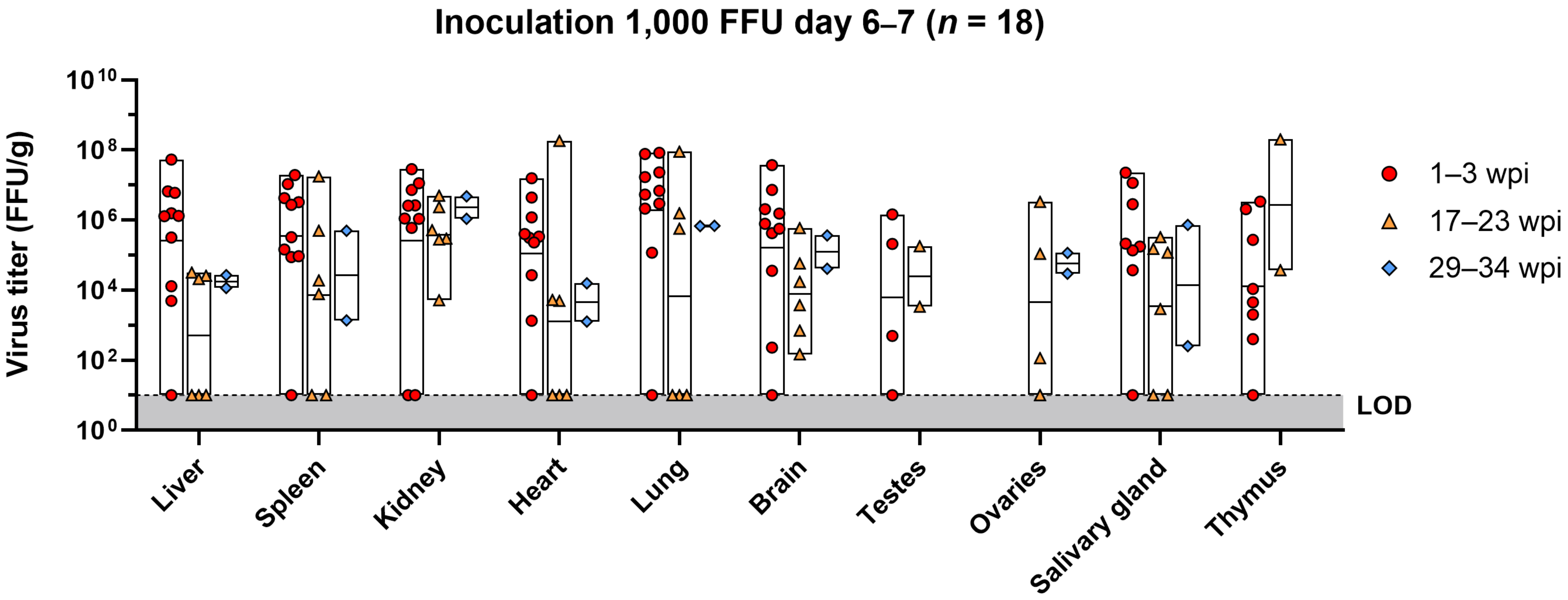

Supplementary Fig. 2. Virus titers in organs of animals inoculated at 6–7 days of age. Animals (*n* = 18) were inoculated s.c. with 1,000 FFU of LASV Ba366. Organs were sampled at different time points and tested for infectious virus via immunofocus assay. Organ samples are depicted in different colors for the different sampling periods (wpi, weeks post-inoculation). All samples falling into the respective time periods have been pooled. Virus titers in organs are shown as bar graphs with all data points shown, the line indicates the mean. The limit of detection (LOD) is indicated by the dotted line and dark grey coloration. Negative samples have been assigned a default value at the limit of detection (10^1 FFU/g). Source data are provided as a Source Data file.

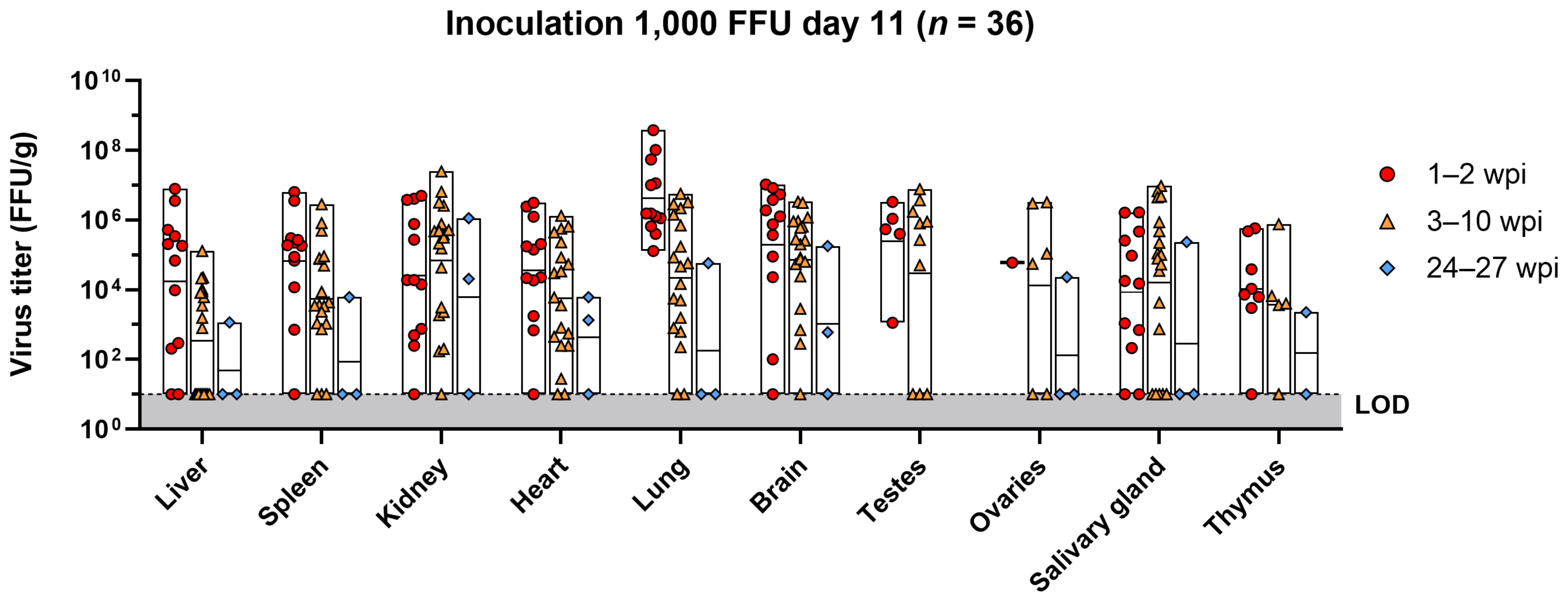

Supplementary Fig. 3. Virus titers in organs of animals inoculated at 11 days of age. Animals (*n* = 36) were inoculated s.c. with 1,000 FFU of LASV Ba366. Organs were sampled at different time points and tested for infectious virus via immunofocus assay. Organ samples are depicted in different colors for the different sampling periods (wpi, weeks post-inoculation). All samples falling into the respective time periods have been pooled. Virus titers in organs are shown as bar graphs with all data points shown, the line indicates the mean. The limit of detection (LOD) is indicated by the dotted line and dark grey coloration. Negative samples have been assigned a default value at the limit of detection (10^1 FFU/g). Source data are provided as a Source Data file.

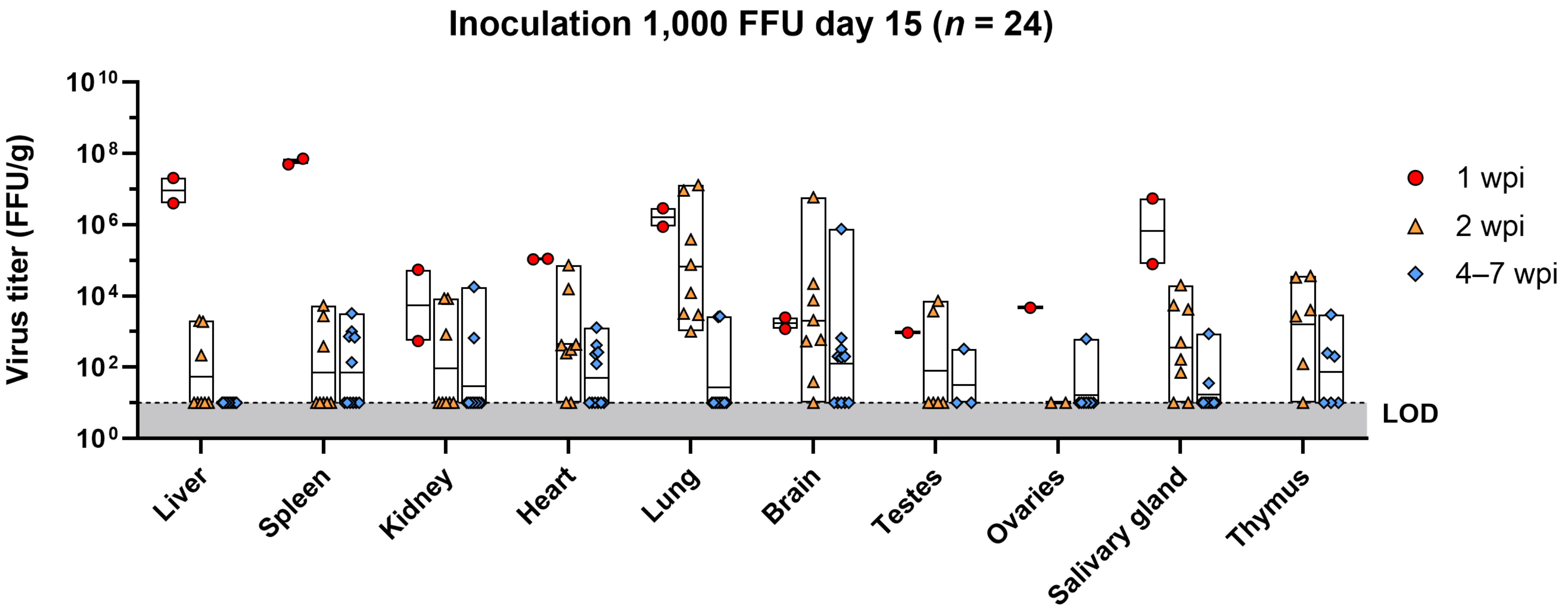

Supplementary Fig. 4. Virus titers in organs of animals inoculated at 15 days of age. Animals (*n* = 24) were inoculated s.c. with 1,000 FFU of LASV Ba366. Organs were sampled at different time points and tested for infectious virus via immunofocus assay. Organ samples are depicted in different colors for the different sampling periods (wpi, weeks post-inoculation). All samples falling into the respective time periods have been pooled. Virus titers in organs are shown as bar graphs with all data points shown, the line indicates the mean. The limit of detection (LOD) is indicated by the dotted line and dark grey coloration. Negative samples have been assigned a default value at the limit of detection (10^1 FFU/g). Source data are provided as a Source Data file.

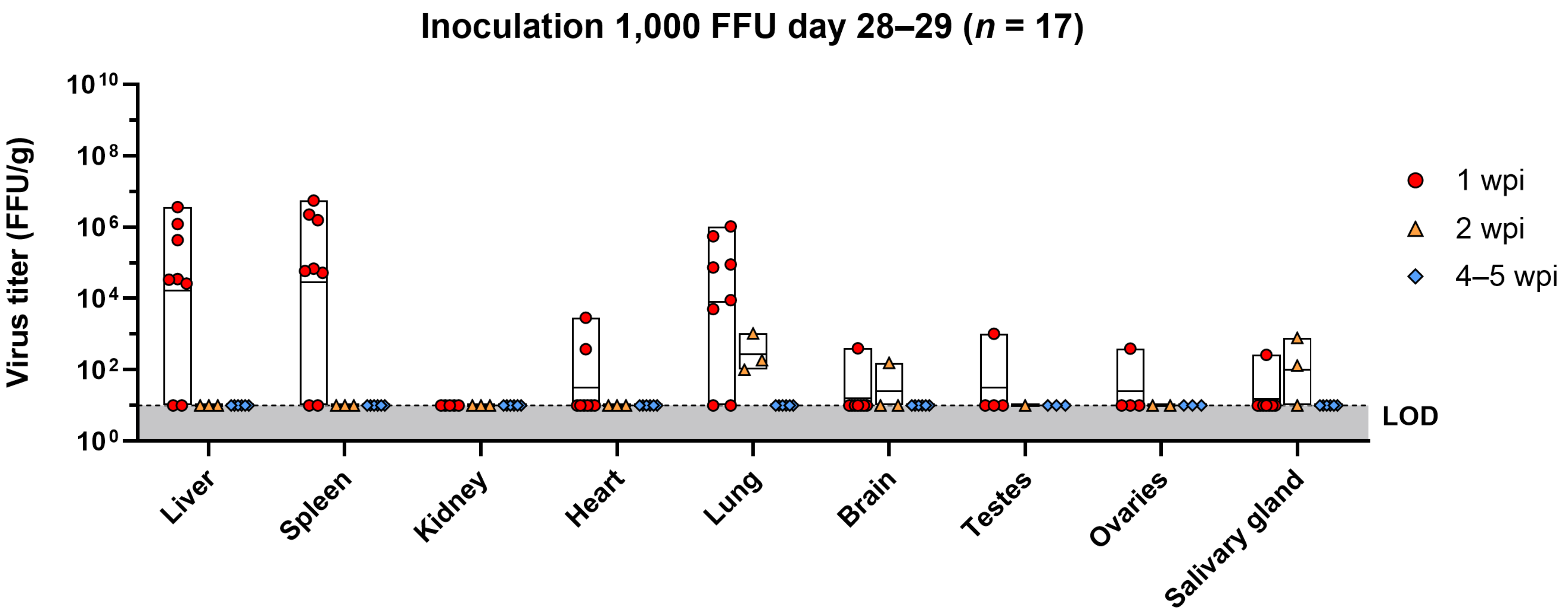

Supplementary Fig. 5. Virus titers in organs of animals inoculated at 28–29 days of age. Animals (*n* = 17) were inoculated s.c. with 1,000 FFU of LASV Ba366. Organs were sampled at different time points and tested for infectious virus via immunofocus assay. Organ samples are depicted in different colors for the different sampling periods (wpi, weeks post-inoculation). All samples falling into the respective time periods have been pooled. Virus titers in organs are shown as bar graphs with all data points shown, the line indicates the mean. The limit of detection (LOD) is indicated by the dotted line and dark grey coloration. Negative samples have been assigned a default value at the limit of detection (10^1 FFU/g). Source data are provided as a Source Data file.

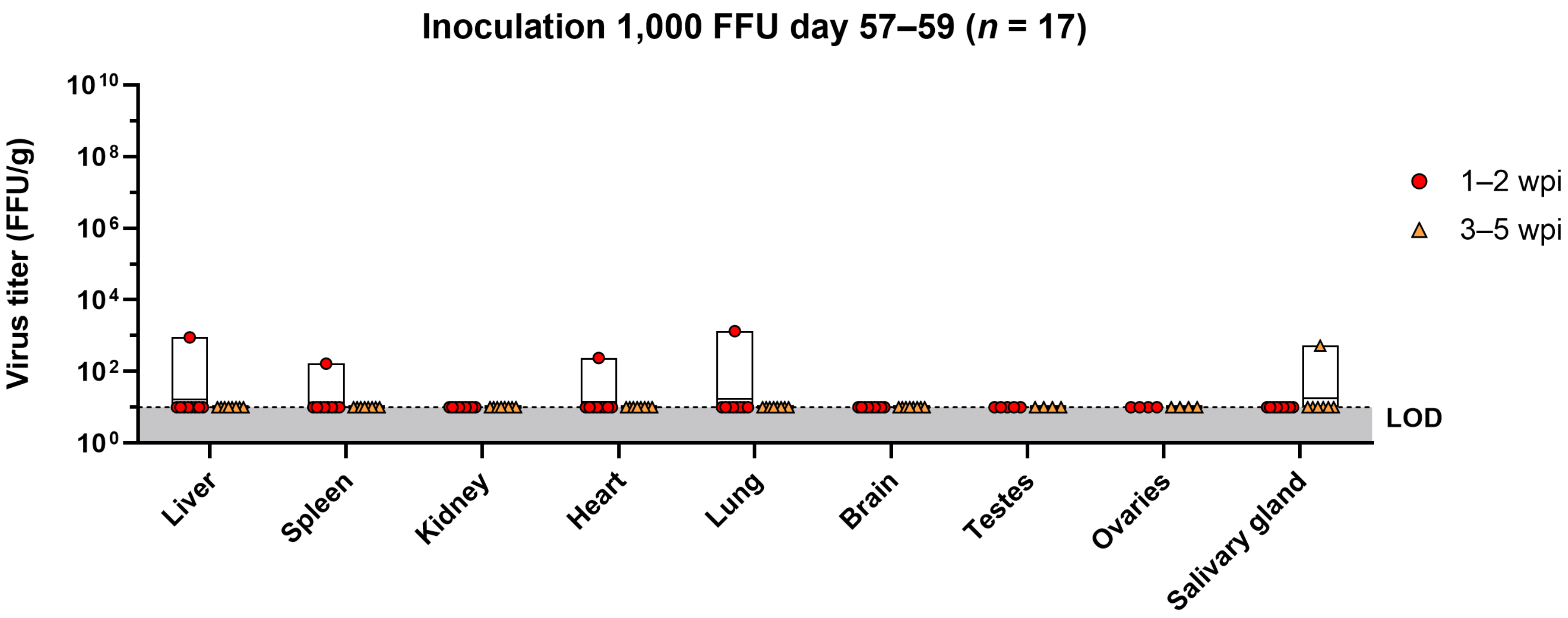

Supplementary Fig. 6. Virus titers in organs of animals inoculated at 57–59 days of age. Animals (*n* = 17) were inoculated s.c. with 1,000 FFU of LASV Ba366. Organs were sampled at different time points and tested for infectious virus via immunofocus assay. Organ samples are depicted in different colors for the different sampling periods (wpi, weeks post-inoculation). All samples falling into the respective time periods have been pooled. Virus titers in organs are shown as bar graphs with all data points shown, the line indicates the mean. The limit of detection (LOD) is indicated by the dotted line and dark grey coloration. Negative samples have been assigned a default value at the limit of detection (10^1 FFU/g). Source data are provided as a Source Data file.

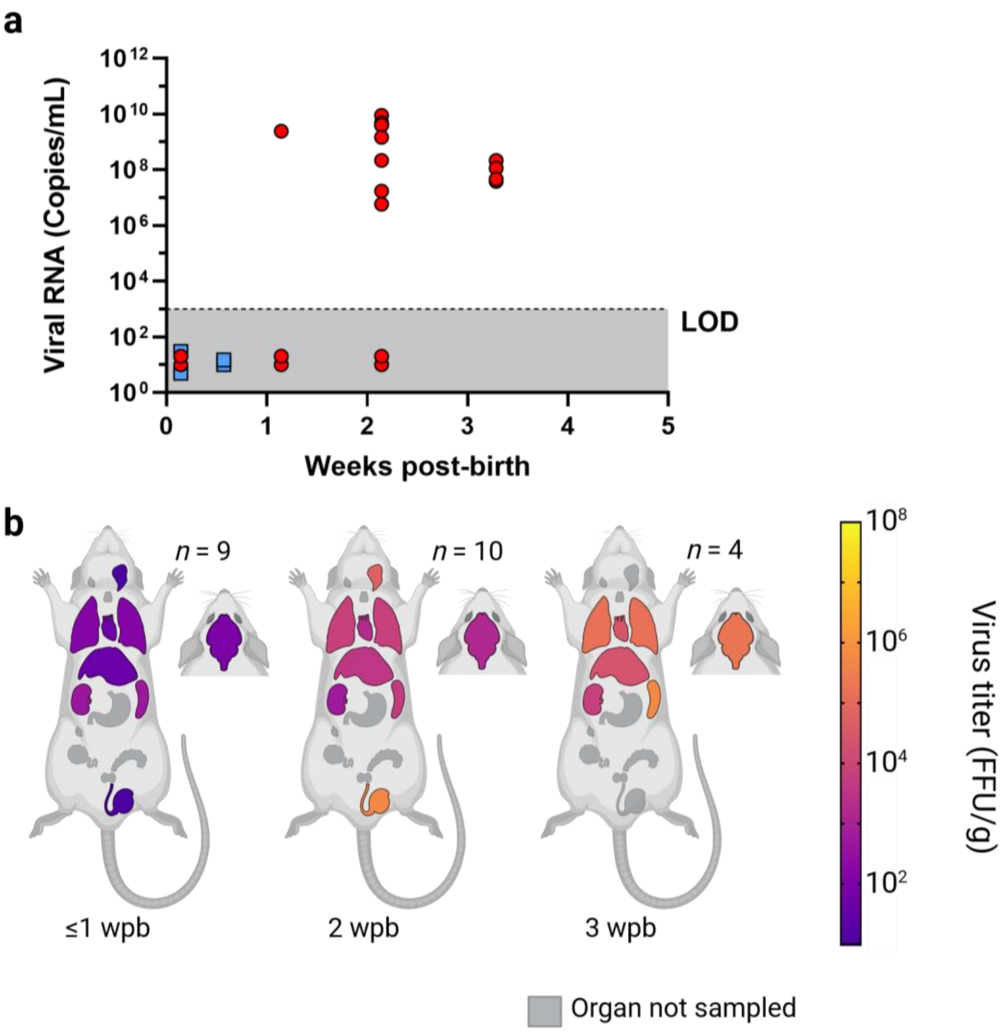

Supplementary Fig. 7. Virus titers in blood and organs of Mastomys exposed to LASV-infected individuals. Neonates (n = 23) were exposed since birth to previously inoculated older siblings (2–3 wpi). Virus RNA titers in blood (copies/mL) and serological status are shown over time (**a**). Blood was sampled at regular intervals and tested for the presence of LASV RNA with qRT-PCR. Ct values were converted into copy numbers using a standard curve. Plasma was inactivated and analyzed for the presence of LASV-specific anti-NP IgG antibodies with ELISA. IgG-positive blood samples are indicated by red dots and IgG-negative samples are depicted as blue squares. Virus titers in organs (**b**) were determined by immunofocus assay. The number of animals euthanized for organ collection (n) is shown for each sampling period (weeks post-birth). Organs are shown as schematic (from cranial to caudal): eyes, brain, salivary glands, cervical lymphnodes, thymus, lung, heart, liver, spleen, kidneys, stomach, inguinal lymphnodes, ovaries, seminal glands, prostate, and testes. Geometric means of LASV-titers in organs are depicted as spectral heat-map. Dark purple: Limit of detection, negative samples have been assigned a default value at the limit of detection. 10^1 FFU/g; yellow: 10^8 FFU/g; Black: All samples in the time period tested negative; Grey: Organ not sampled. The limit of detection (LOD) for the qRT-PCR assay is indicated by the dotted line and dark grey colouration. PCR-negative samples have been assigned a default value below the limit of detection. Source data are provided as a Source Data file. Created in BioRender. Oestereich, L. (2024) BioRender.com/i19v220.

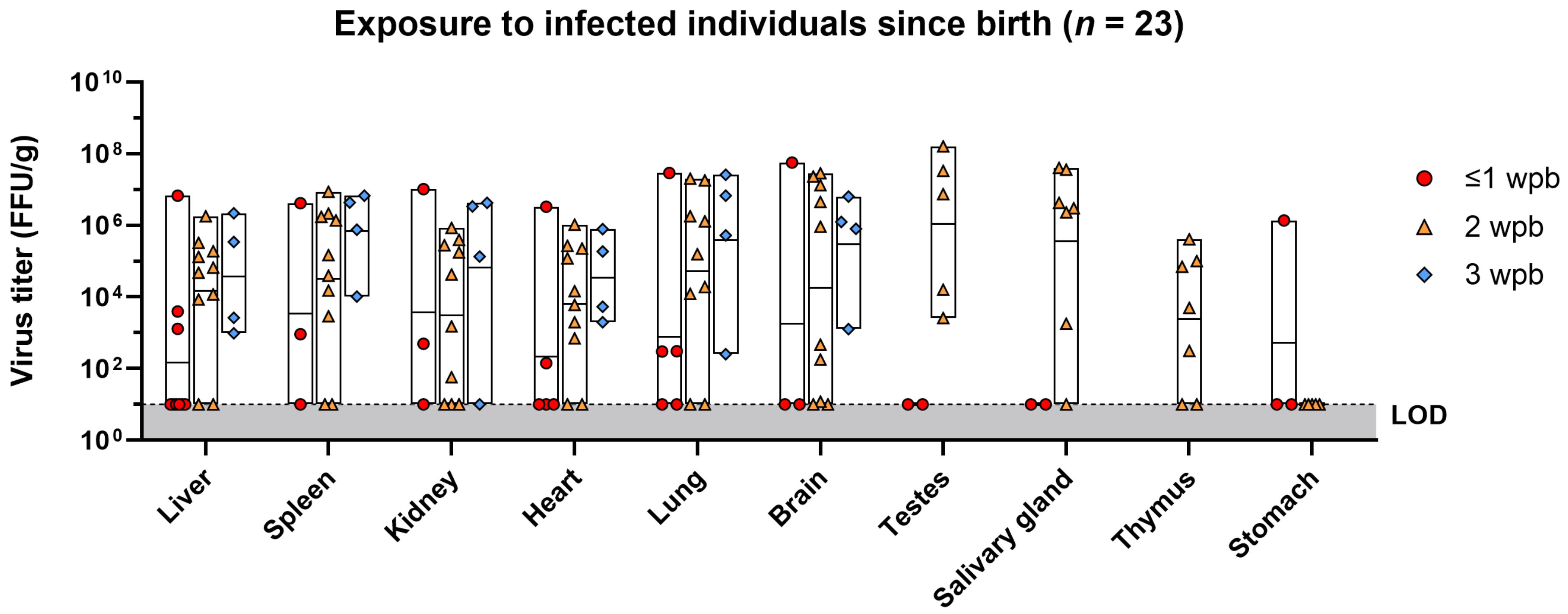

Supplementary Fig. 8. Virus titers in organs of animals exposed to LASV-infected individuals since birth. Animals (*n* = 23) were co-housed with previously inoculated (LASV Ba366; 2–3 wpi) older siblings since birth. Organs were sampled at different time points and tested for infectious virus via immunofocus assay. Organ samples are depicted in different colors for the different sampling periods (wpb, weeks post-birth). All samples falling into the respective time periods have been pooled. Virus titers in organs are shown as bar graphs with all data points shown, the line indicates the mean. The limit of detection (LOD) is indicated by the dotted line and dark grey coloration. Negative samples have been assigned a default value at the limit of detection (10^1 FFU/g). Source data are provided as a Source Data file.

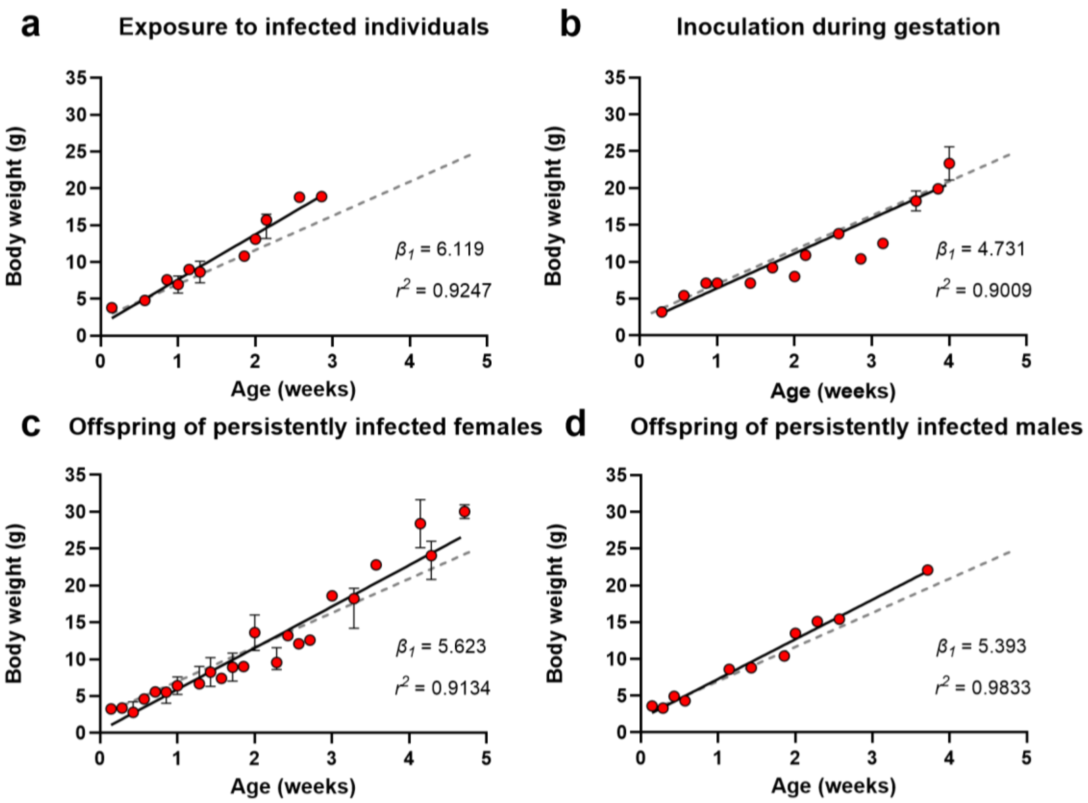

Supplementary Fig. 9. Growth and development of Mastomys exposed or borne to infected individuals. The body weight of animals exposed to previously inoculated older siblings (2–3 wpi) (**a**, *n* = 23), borne to females inoculated during gestation (**b**, *n* = 27), borne to persistently infected females (**c**, *n* = 149), or fathered by persistently infected males (**d**, *n* = 92) has been measured during the first four weeks of life. The body weight shown as median with error is depicted as red dots. The weight gain in gram per week (β_1) for each group was determined by simple linear regression. The linear regression line of the naïve control group is depicted in grey. No significant differences in the weight gain of inoculated individuals compared to the naïve control were detected, as determined by one-way ANOVA and *Dunnett's multiple comparison*. Source data are provided as a Source Data file.

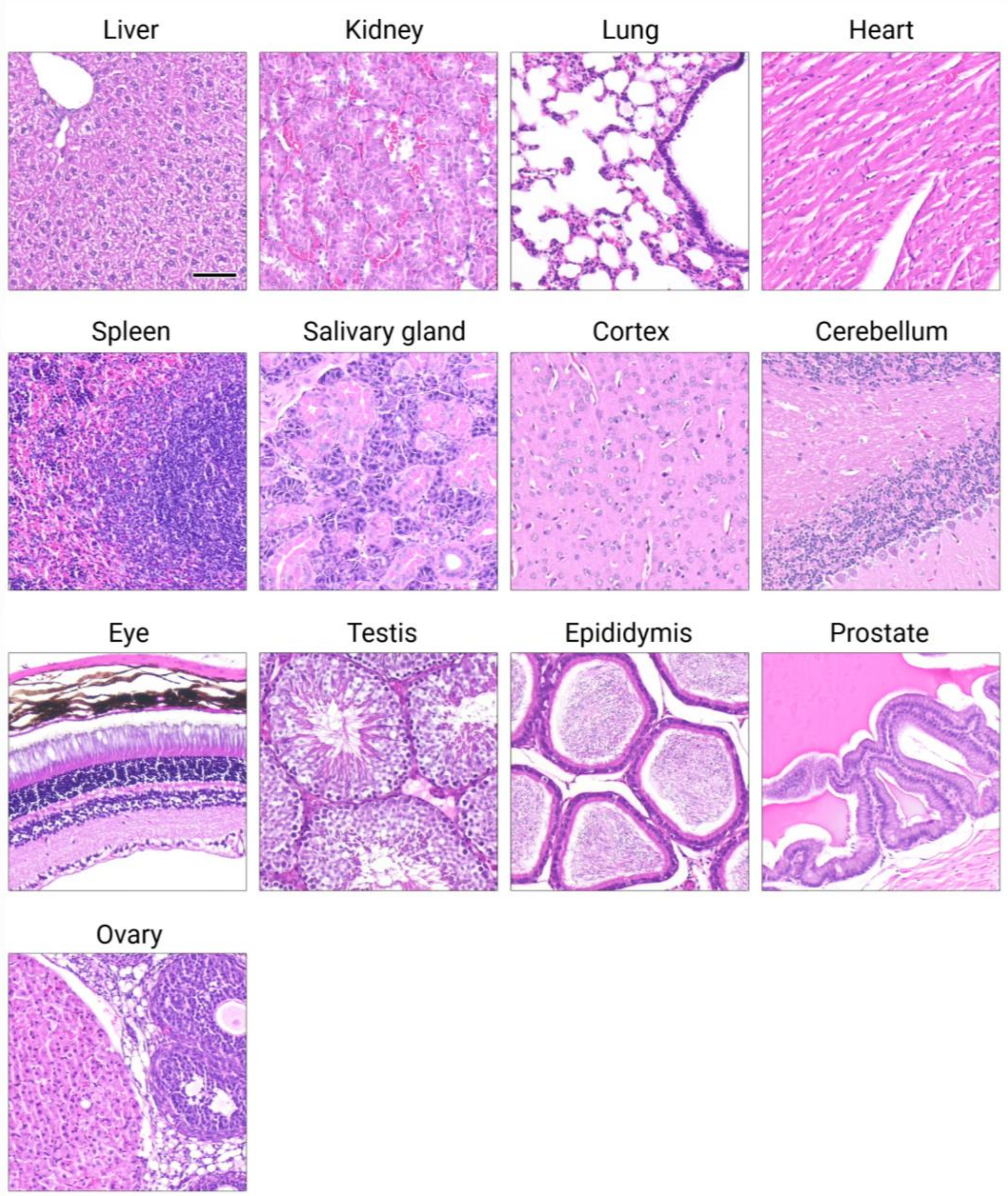

Supplementary Fig. 10. Histological analysis of organs following prenatal infection with LASV. Pregnant females were inoculated i.v. with 10,000 FFU of LASV Ba366 approximately two weeks into gestation. Organs of female and male offspring (*n* = 4) were analyzed in histopathology. Infected animals (≥245 days post-birth) displayed no signs of immune infiltration, cell loss, or degeneration. Representative images of H&E-stained tissue sections are shown (from left to right): liver, kidney, lung, heart, spleen, salivary gland, cortex, cerebellum, eye, testis, epididymis, prostate, and ovary. Black scale bar equals 50 µm.

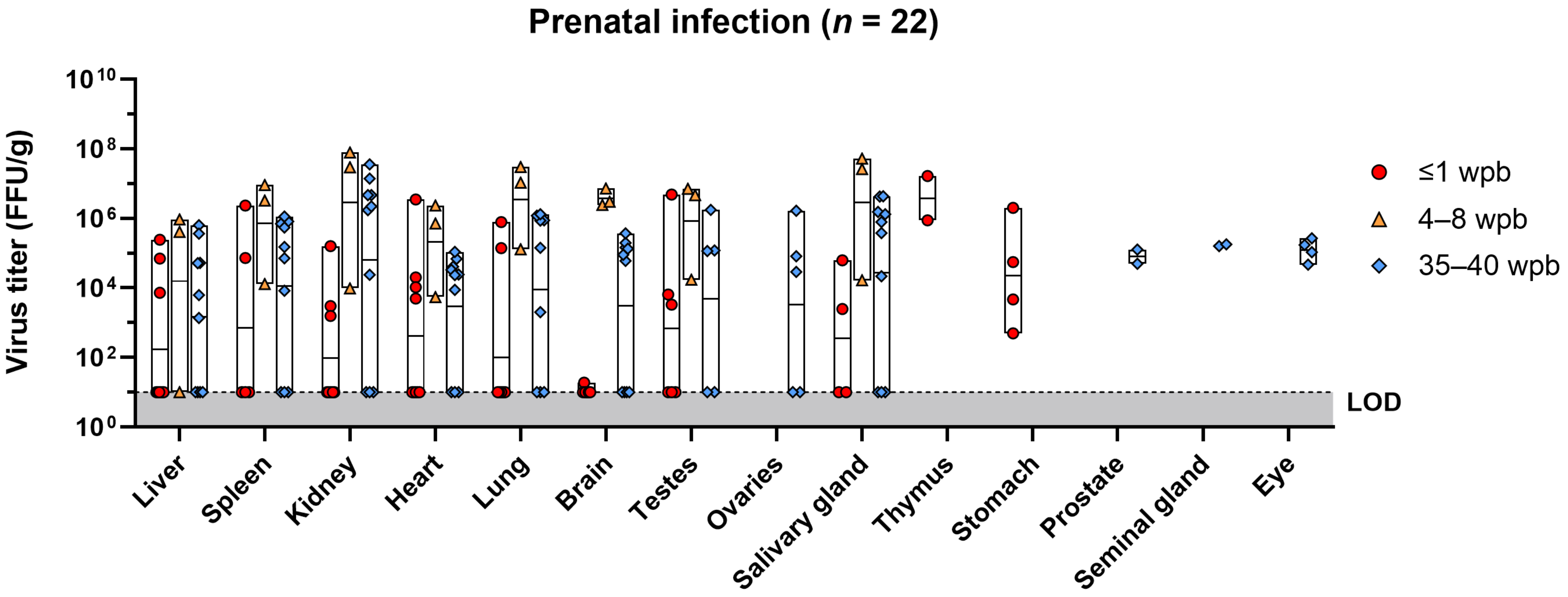

Supplementary Fig. 11. Virus titers in organs of animals following prenatal infection. Pregnant females were inoculated i.v. with 10,000 FFU of LASV Ba366 roughly two weeks into gestation. Organs of the offspring (*n* = 27) borne to the inoculated females were tested for the presence of LASV. Organs were sampled at different time points and tested for infectious virus via immunofocus assay. Organ samples are depicted in different colors for the different sampling periods (wpb, weeks post-birth). All samples falling into the respective time periods have been pooled. Virus titers in organs are shown as bar graphs with all data points shown, the line indicates the mean. The limit of detection (LOD) is indicated by the dotted line and dark grey coloration. Negative samples have been assigned a default value at the limit of detection (10^1 FFU/g). Source data are provided as a Source Data file.

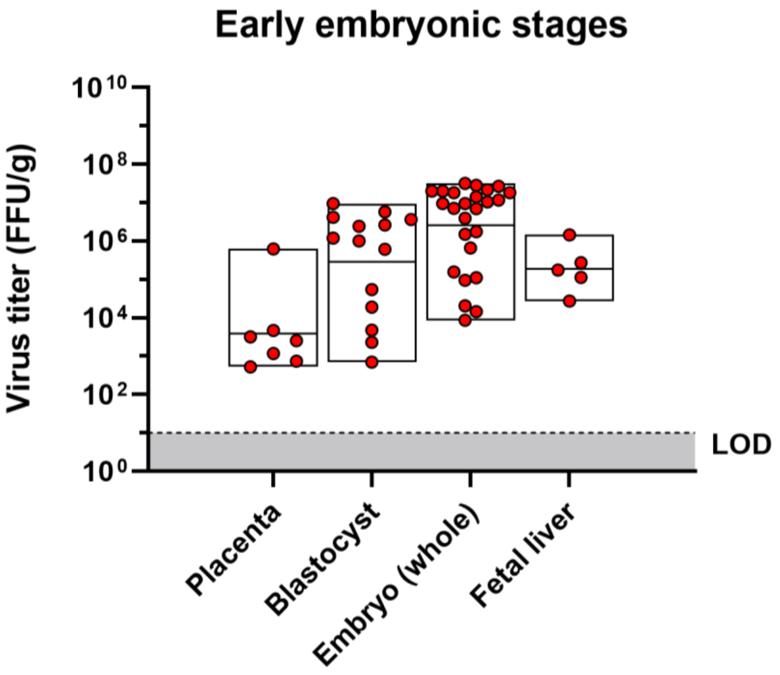

Supplementary Fig. 12. Virus titers in placentas and embryos collected from persistently infected females. Placentas (*n* = 7), blastocysts (*n* = 14), whole embryos (*n* = 25), and fetal livers (*n* = 5) were tested for LASV presence via immunofocus assay. Virus titers in organs are shown as bar graphs with all data points shown, the line indicates the mean. The limit of detection (LOD) is indicated by the dotted line and dark grey coloration. Negative samples have been assigned a default value at the limit of detection (10^1 FFU/g). Source data are provided as a Source Data file.

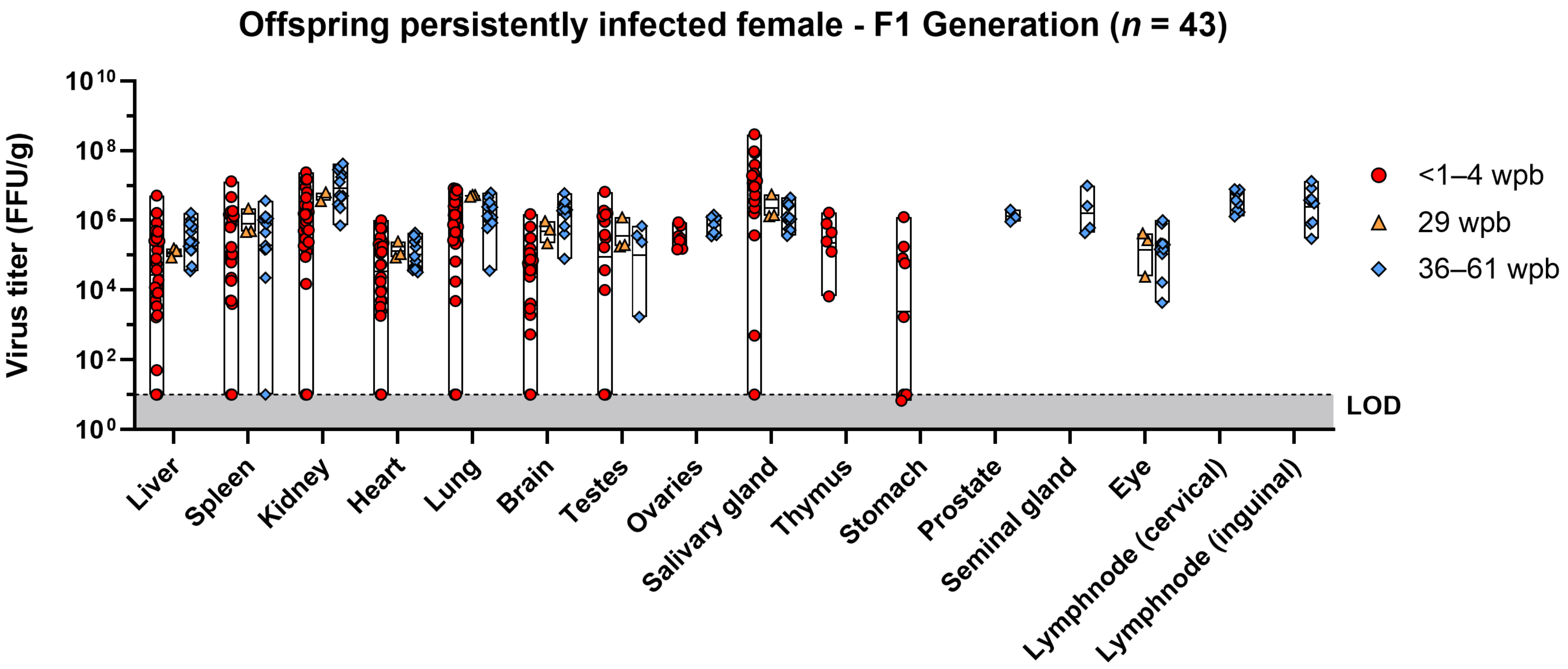

Supplementary Fig. 13. Virus titers in organs of animals born to persistently infected females (F1 Generation). Persistently infected females were bred with naïve males. Females from the resulting litters were further bred. LASV was passaged across five filial generations (F1 to F5) using persistently infected females. Organs of the first three generations were collected and tested for the presence of LASV. Organs of the F1 generation (*n* = 43) were sampled at different time points and tested for infectious virus via immunofocus assay. Organ samples are depicted in different colors for the different sampling periods (wpb, weeks post-birth). All samples falling into the respective time periods have been pooled. Virus titers in organs are shown as bar graphs with all data points shown, the line indicates the mean. The limit of detection (LOD) is indicated by the dotted line and dark grey coloration. Negative samples have been assigned a default value at the limit of detection (10^1 FFU/g). Source data are provided as a Source Data file.

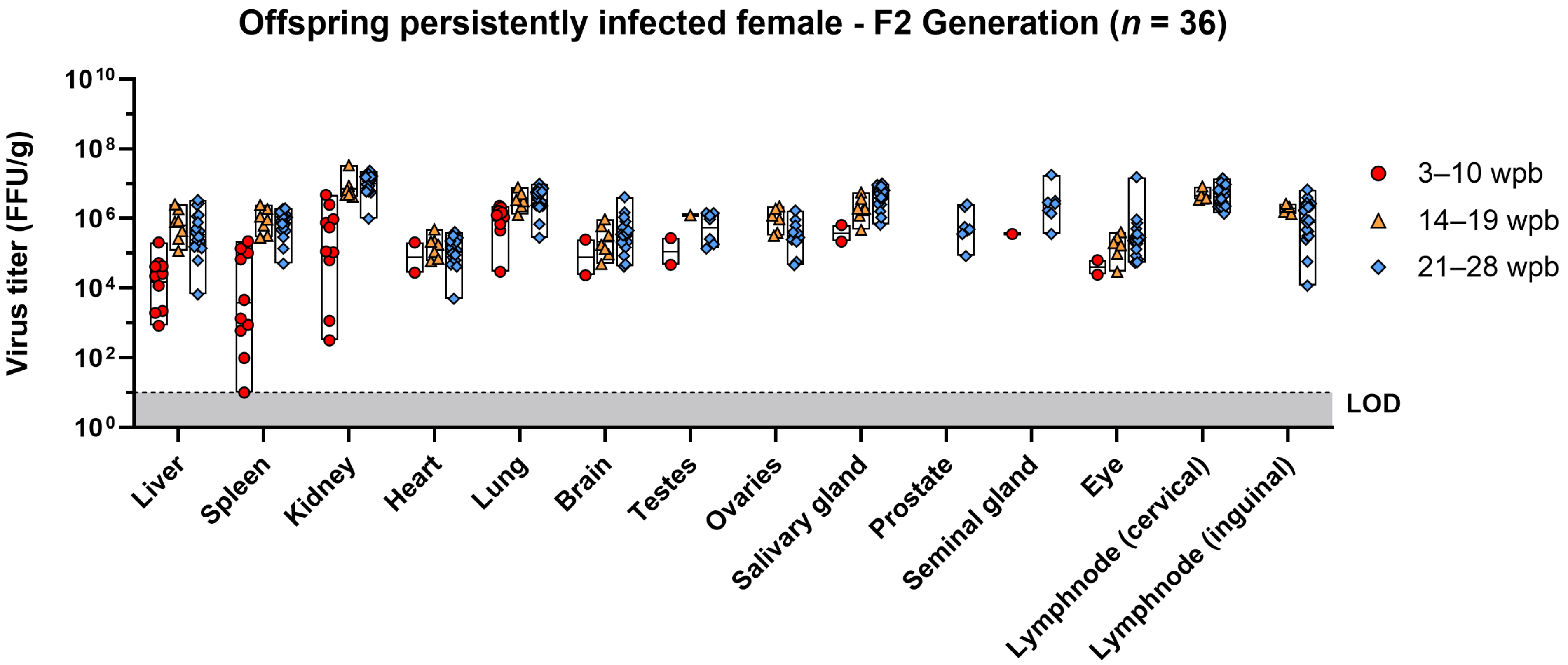

Supplementary Fig. 14. Virus titers in organs of animals born to persistently infected females (F2 Generation). Persistently infected females were bred with naïve males. Females from the resulting litters were further bred. LASV was passaged across five filial generations (F1 to F5) using persistently infected females. Organs of the first three generations were collected and tested for the presence of LASV. Organs of the F2 generation (*n* = 36) were sampled at different time points and tested for infectious virus via immunofocus assay. Organ samples are depicted in different colors for the different sampling periods (wpb, weeks post-birth). All samples falling into the respective time periods have been pooled. Virus titers in organs are shown as bar graphs with all data points shown, the line indicates the mean. The limit of detection (LOD) is indicated by the dotted line and dark grey coloration. Negative samples have been assigned a default value at the limit of detection (10^1 FFU/g). Source data are provided as a Source Data file.

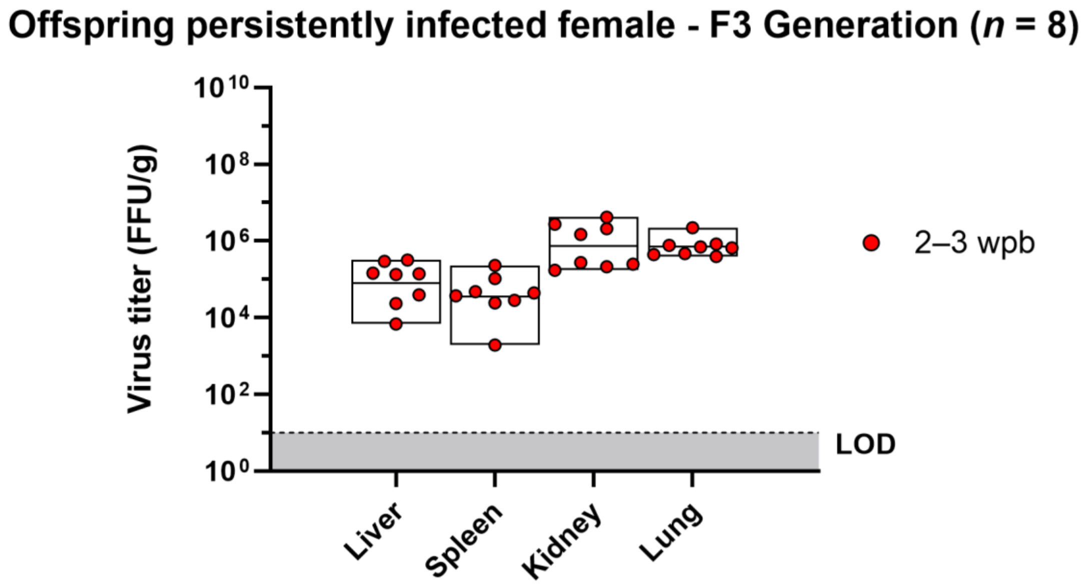

Supplementary Fig. 15. Virus titers in organs of animals born to persistently infected females (F3 Generation). Persistently infected females were bred with naïve males. Females from the resulting litters were further bred. LASV was passaged across five filial generations (F1 to F5) using persistently infected females. Organs of the first three generations were collected and tested for the presence of LASV. Organs of the F3 generation (*n* = 8) were sampled at different time points and tested for infectious virus via immunofocus assay. were collected at 2–3 weeks post-birth (wpb). All samples falling into this time period have been pooled. Virus titers in organs are shown as bar graphs with all data points shown, the line indicates the mean. The limit of detection (LOD) is indicated by the dotted line and dark grey coloration. Negative samples have been assigned a default value at the limit of detection (10^1 FFU/g). Source data are provided as a Source Data file.

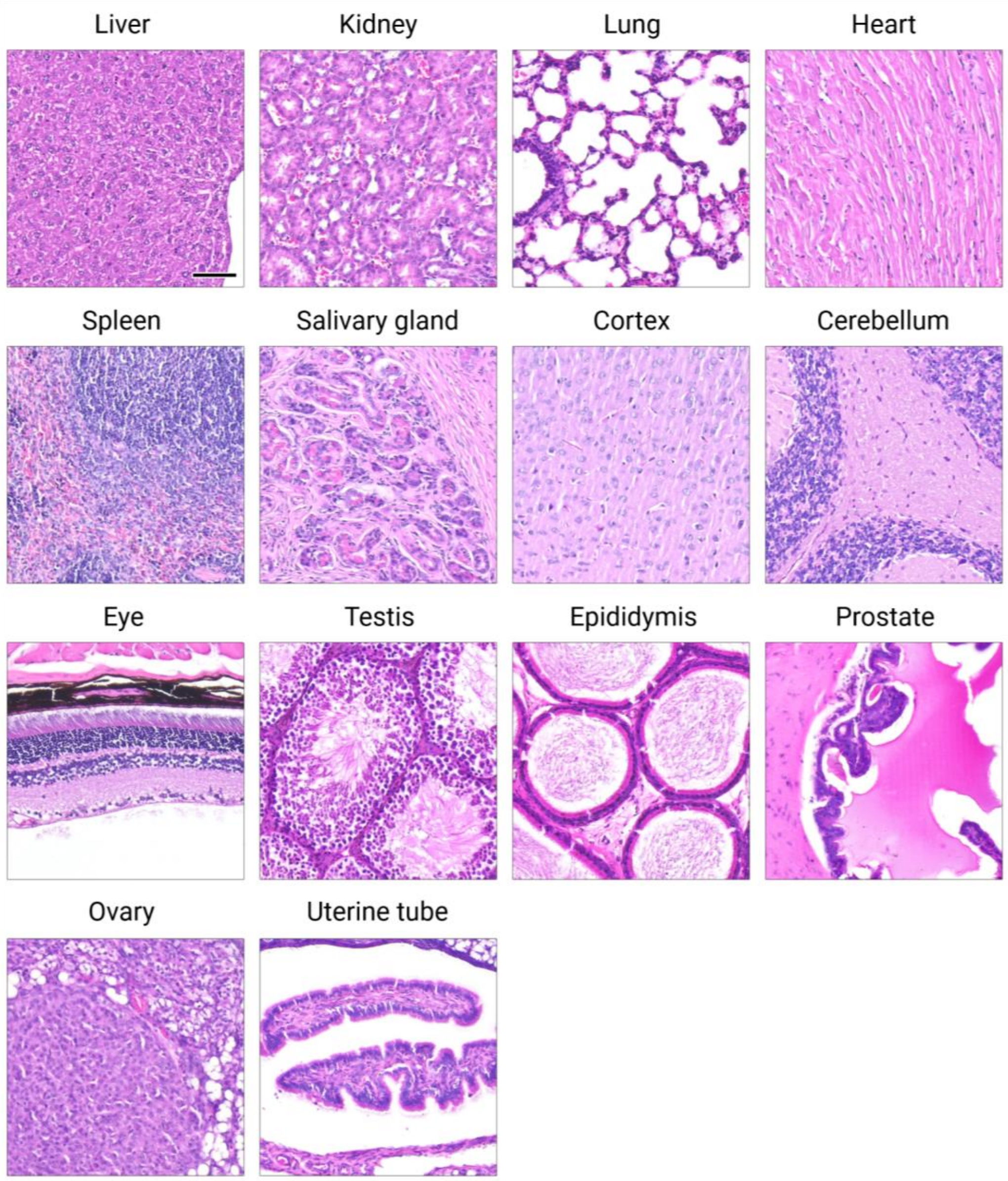

Supplementary Fig. 16. Histological analysis of organs from animals borne to persistently infected females. Persistently infected females were bred with naïve males. Organs of female and male offspring (*n* = 27) were analyzed. Infected animals (≥159 days post-birth) displayed no signs of immune infiltration, cell loss, or degeneration. Representative images of H&E-stained tissue sections are shown (from left to right): liver, kidney, lung, heart, spleen, salivary gland, cortex, cerebellum, eye, testis, epididymis, prostate, ovary, and uterine tube. Black scale bar equals 50 µm.

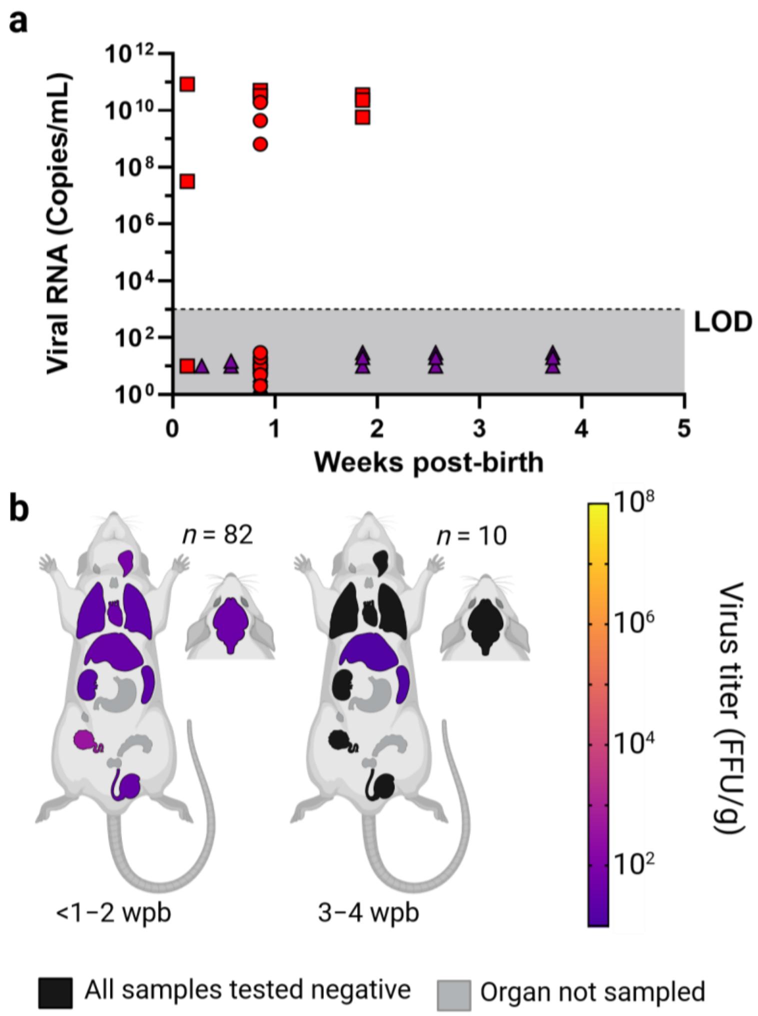

Supplementary Fig. 17. Virus titers in blood and organs of Mastomys fathered by LASV-infected males. Persistently infected males were bred with naive females. Pregnant females were separated from their mate roughly two weeks into gestation. The resulting offspring (n = 92) never had direct or indirect contact with their fathers except through their mother. Data is shown for litters with at least one LASV-positive (blood and/or organs) individual. Virus RNA titers in blood (copies/mL) are shown over time (**a**). Blood was sampled at regular intervals and tested for the presence of LASV RNA with qRT-PCR. Ct values were converted into copy numbers using a standard curve. Plasma was inactivated and analyzed for the presence of LASV-specific anti-NP IgG antibodies with ELISA. Samples from the two viremic litters are depicted in red. Litters were considered viremic if at least one individual tested positive for LASV in blood. Samples from non-viremic litters that showed LASV-presence in organs, depicted as purple triangles, were pooled. All tested animals were IgG-positive. Virus titers in organs (**b**) were determined by immunofocus assay. The number of animals euthanized for organ collection (n) is shown for each sampling period (weeks post-birth). Organs are shown as schematic (from cranial to caudal): eyes, brain, salivary glands, cervical lymphnodes, thymus, lung, heart, liver, spleen, kidneys, stomach, inguinal lymphnodes, ovaries, seminal glands, prostate, and testes. Geometric means of LASV-titers in organs are depicted as spectral heat-map. Dark purple: Limit of detection, negative samples have been assigned a default value at the limit of detection. 10^{1} FFU/g; yellow: 10^{8} FFU/g; Black: All samples in the time period tested negative; Grey: Organ not sampled. The limit of detection (LOD) for the qRT-PCR assay is indicated by the dotted line and dark grey coloration. PCR-negative samples have been assigned a default value below the limit of detection. Source data are provided as a Source Data file. Created in BioRender. Oestereich, L. (2024) BioRender.com/i19v220.

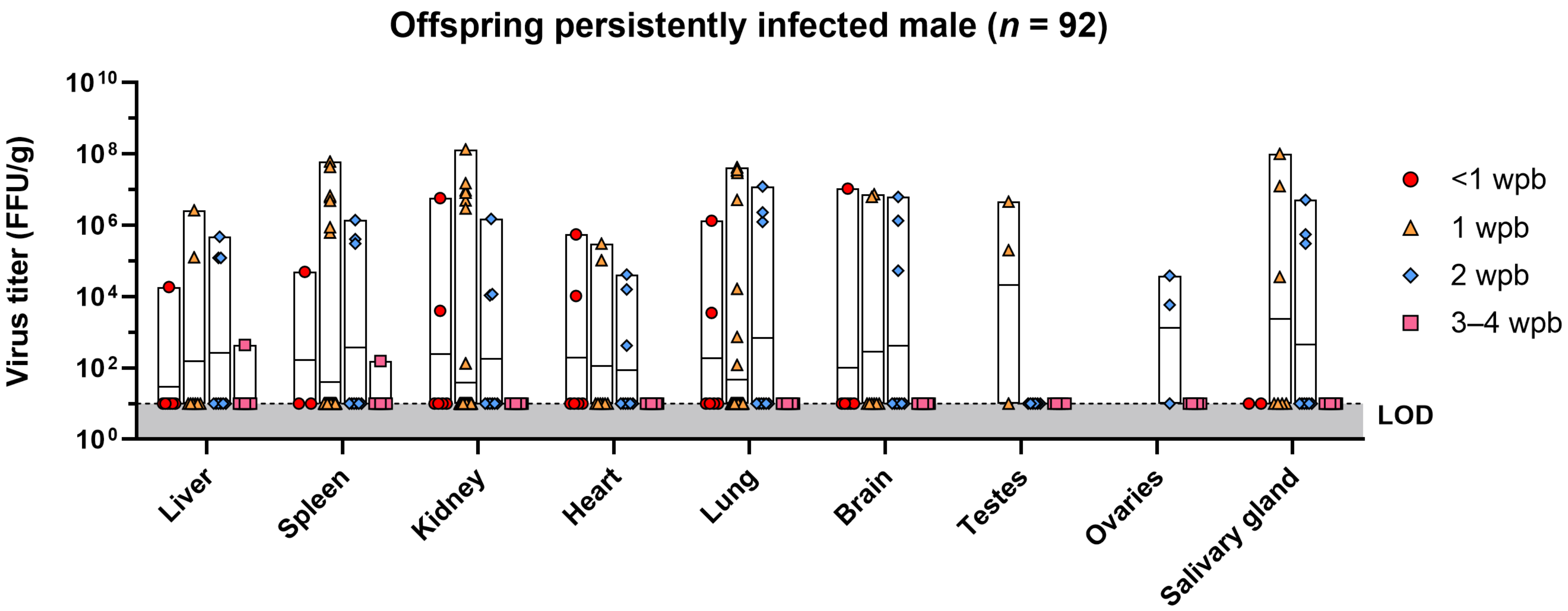

Supplementary Fig. 18. Virus titers in organs of animals fathered by persistently infected males. Persistently infected males were bred with naïve males. Pregnant females were separated from their mate roughly two weeks into gestation. The resulting offspring (*n* = 92) never had direct or indirect contact with their fathers except through their mother. Organs were sampled at different time points and tested for infectious virus via immunofocus assay. Organ samples are depicted in different colors for the different sampling periods (wpb, weeks post-birth). All samples falling into the respective time periods have been pooled. Virus titers in organs are shown as bar graphs with all data points shown, the line indicates the mean. The limit of detection (LOD) is indicated by the dotted line and dark grey coloration. Negative samples have been assigned a default value at the limit of detection (10^1 FFU/g). Source data are provided as a Source Data file.

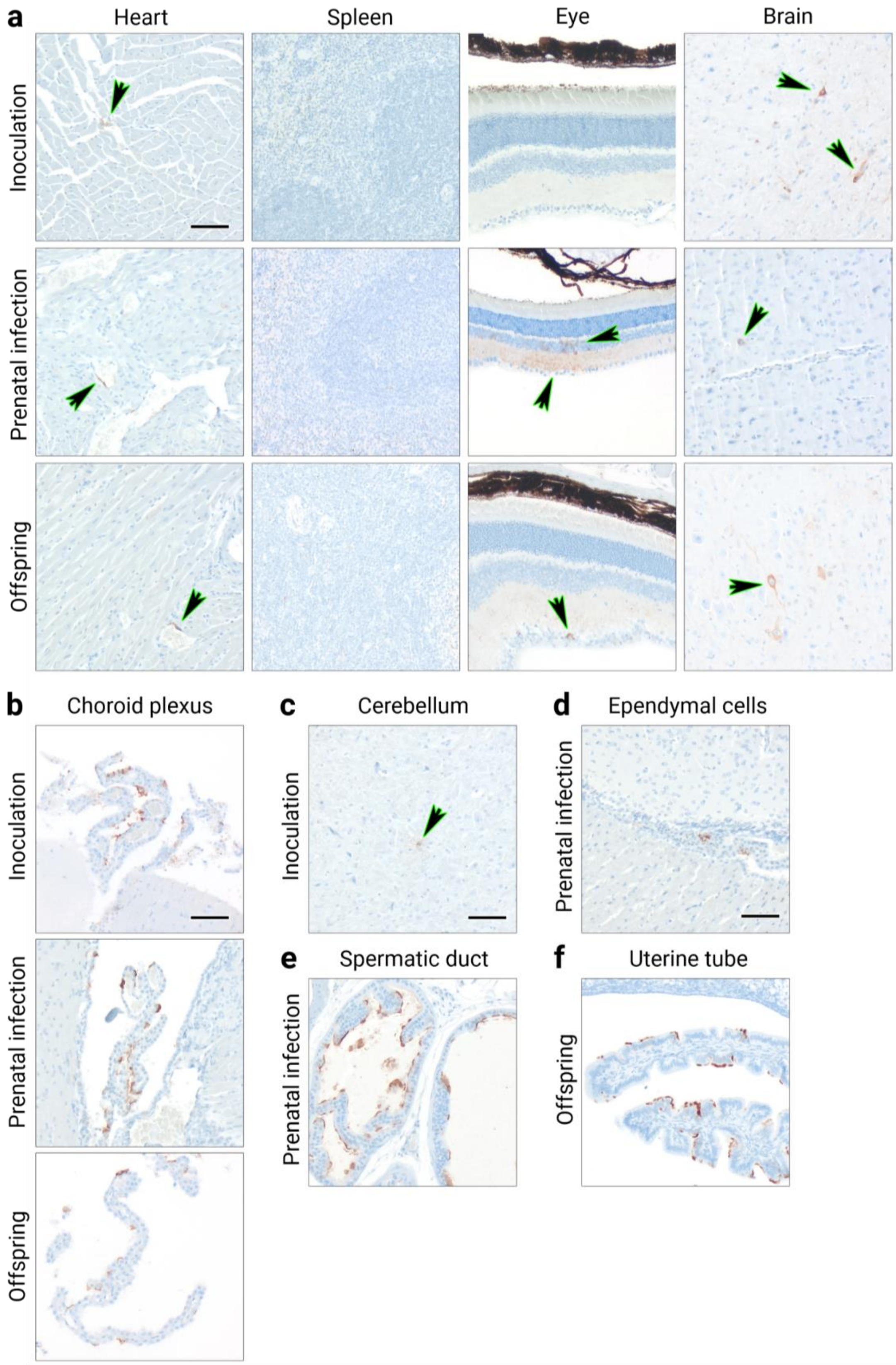

Supplementary Fig. 19. LASV in organs of persistently infected animals. Animals were either inoculated s.c. with 1,000 FFU of LASV Ba366 at 6–7 days of age (inoculation, *n* = 5), borne to females inoculated during gestation (prenatal infection, *n* = 4), or borne to persistently infected females (offspring, *n* = 27). Organs were sampled from persistently infected individuals (stable virus titers in blood for ≥120 days). Immunohistochemical analysis of heart spleen, eye, and brain (**a**) shows LASV presence in vascular endothelial cells, the plexiform and inner granular layer, as well as ganglion cells of the retina. Virus positive neurons and endothelial cells were found in the brain. No positive signal could be detected in spleens. Epithelial cells in the choroid plexus (**b**), oligodendrocytes in the cerebellum (**c**), ependymal cells (**d**), as well as epithelial cells in the spermatic duct (**e**) and uterine tube (**f**) were

also infected with LASV. Representative images of the immunohistochemical analysis are shown. Arrows serve as visual aid indicating infected cells. Black scale bar equals 50 µm.

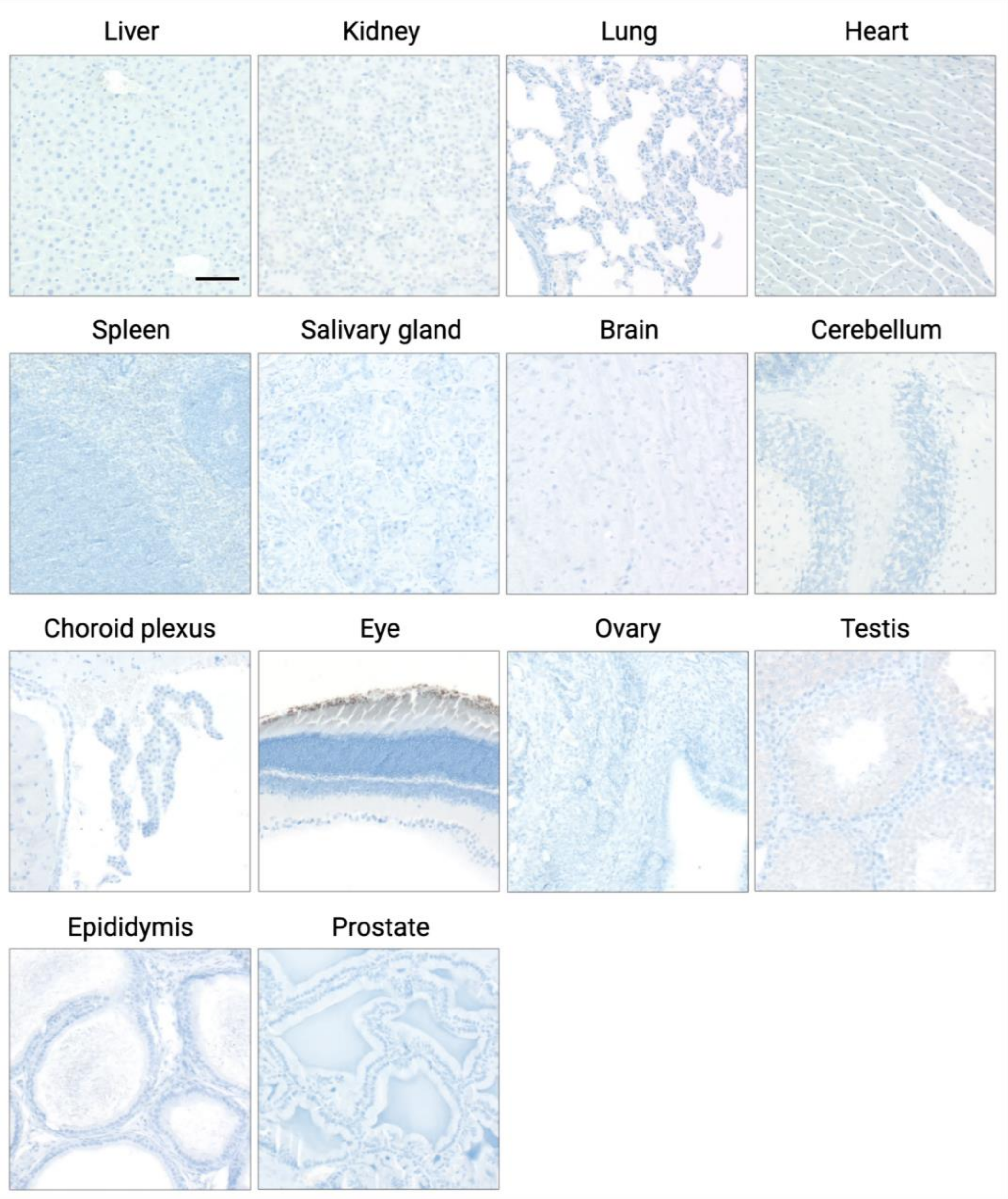

Supplementary Fig. 20. Immunohistochemical analysis of naïve control animals. Organs of naïve Mastomys (*n* = 2) were also stained for LASV glycoprotein. No positive signal was detected in the control group. Representative images of the immunohistochemical analysis are shown. Black scale bar equals 50 µm.

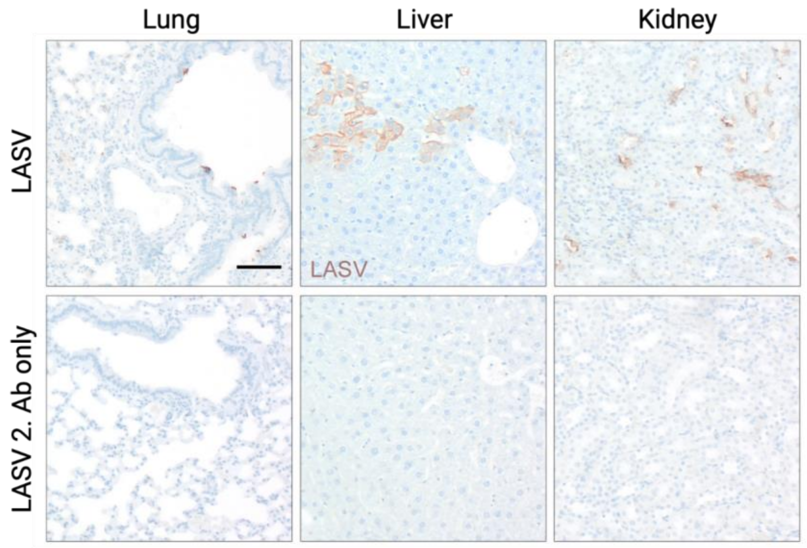

Supplementary Fig. 21. Antibody staining control. Lung, liver, and kidney from animals inoculated with 1,000 FFU of LASV Ba366 at 6–7 days of age showing specific staining for LASV compared to the staining controls only treated with the secondary antibody. Representative images of the immunohistochemical analysis are shown. Black scale bar equals 50 µm.

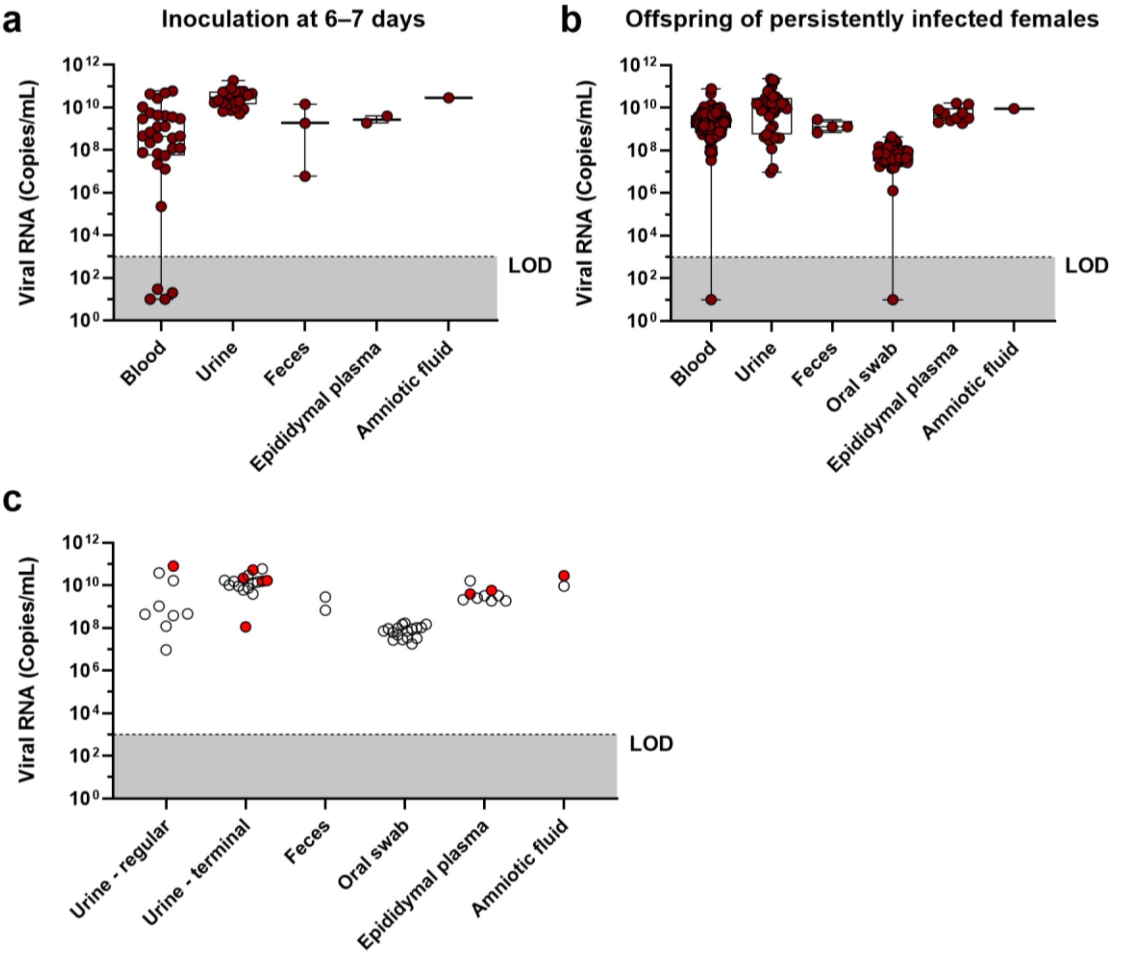

d

Urine		Feces	Oral swab	Epididymal plasma	Amniotic fluid
Regular[1]	Terminal[2]				
1/9	5/16	0/2	0/17	2/9	1/2

Supplementary Fig. 22. LASV in body fluids and excretions. Virus RNA titers (copies/mL) in blood, urine, feces, oral swabs, epididymal plasma, and amniotic fluid of animals inoculated with 1,000 FFU of LASV Ba366 at 6–7 days of age (**a**) or animals borne to persistently infected females (**b**). Body fluid samples were tested for the presence of LASV RNA with qRT-PCR. Ct values were converted into copy numbers using a standard curve. Selected samples from both groups were used for virus isolation (**c**). Samples where virus isolation was successful are depicted as bright red dots and samples where isolation attempts failed are indicated by white dots. The limit of detection (LOD) for the qRT-PCR assay is shown by the dotted line and dark grey coloration. Results of the in vitro isolations from collected body fluids are summarized in the table (**d**). Given are the numbers of successful isolations from all isolation attempts. [1] Regular urine samples have been collected following voiding. [2] Terminal urine samples were acquired via bladder puncture following euthanasia. Source data are provided as a Source Data file.

Supplementary Table 1. Virus RNA presence in body fluids, antibody status, and infectious virus in organs of Mastomys following inoculation with Lassa virus (LASV) strain Ba366.

Group	Sampling Period (Weeks Post-Inoculation)	Blood		Urine	Organs
		PCR Positive Samples/Tested Samples[1] per Sampling Period	Ab Positive Samples/Tested Samples[1] per Sampling Period	PCR Positive Samples/Tested Samples[1] per Sampling Period	Virus Positive Animals/Tested Animals[2] per Sampling Period
Inoculation 1,000 FFU day 6–7 (*n* = 18)	1–3	12/12	5/11	4/4	10/10
	4–8	5/5	5/6	11/11	n.t.
	9–15	5/5	4/4	4/4	n.t.
	17–23	3/7	7/7	4/4	6/6
	29–34	2/2	2/2	n.t.	2/2
Inoculation 1,000 FFU day 11 (*n* = 36)	1–2	12/12	7/12	4/5	12/12
	3–5	10/11	11/11	5/5	8/8
	6–10	10/13	12/13	1/1	11/11
	12–15	2/6	4/5	n.t.	n.t.
	24–27	1/3	3/3	n.t.	3/3
Inoculation 1,000 FFU day 15 (*n* = 24)	1	2/2	0/2	1/1	2/2
	2	8/8	8/8	2/3	8/8
	4–7	10/14	14/14	n.t.	10/11
Inoculation 1,000 FFU day 28–29 (*n* = 17)	1	7/8	0/8	1/3	6/8
	2	1/3	3/3	n.t.	3/3
	4–5	0/6	6/6	n.t.	0/6
Inoculation 1,000 FFU day 57–59 (*n* = 21)	1–3	2/13	12/13	n.t.	3/9
	4–5	0/8	6/8	n.t.	1/8

[1] For blood and urine, the number of positive samples versus the total number of samples tested during a given time period is shown. Since individuals were sampled up to 3 times per sampling period, the number of tested samples can exceed the number of animals per group. [2] For organs, the number of positive animals versus the total number of tested animals is shown. The following organs were sampled: liver, spleen, kidney, heart, lung, gonads, salivary glands and thymus. Animals were considered positive, if one or more of the tested organs contained infectious virus. n.t. = not tested.

Supplementary Table 2. Virus RNA presence in body fluids, antibody status, and infectious virus in organs of Mastomys following exposure to LASV-infected individuals.

Group	Sampling Period (Weeks Post-Birth)	Blood		Urine	Organs
		PCR Positive Samples/Tested Samples[1] per Sampling Period	Ab Positive Samples/Tested Samples[1] per Sampling Period	PCR Positive Samples/Tested Samples[1] per Sampling Period	Virus Positive Animals/Tested Animals[2] per Sampling Period
Exposure to infected individuals since birth[3] (*n* = 23)	≤1	1/9	5/9	n.t.	3/9
	2	8/10	10/10	1/1	8/10
	3	4/4	4/4	2/2	4/4
Exposure of adults to infected individuals[4] (*n* = 33)	16–52	0/33	30/33	n.t.	0/6

[1] For blood and urine, the number of positive samples versus the total number of samples tested during a given time period is shown. Since individuals were sampled up to 3 times per sampling period, the number of tested samples can exceed the number of animals per group. [2] For organs, the number of positive animals versus the total number of tested animals is shown. The following organs were sampled: liver, spleen, kidney, heart, lung, brain, gonads, and salivary glands. Animals were considered positive, if one or more of the tested organs contained infectious virus. n.t. = not tested. [3] Neonates were exposed from birth via co-housing to infected older siblings (2–3 wpi). [4] Naïve adults were exposed to infected individuals from the moment of inoculation of their offspring (at 6–7, 11, or 15 days of age) and remained in direct contact for 1–3 weeks until the weaning of the young at the age of 3–4 weeks. Females that gave birth to another litter remained in contact for 4–6 weeks. Naïve adults that were paired with persistently infected partners remained in direct contact with their mate until the end of the breeding experiments (4–21 weeks).

Supplementary Table 3. Virus RNA presence in body fluids, antibody status, and infectious virus in organs of Mastomys following prenatal infection or of individuals borne to persistently infected parents.

Group		Sampling Period (Weeks Post-Birth)	Blood		Urine	Oral Swab	Organs
			**PCR Positive Samples/Tested Samples[1] per Sampling Period**	**Ab Positive Samples/Tested Samples[1] per Sampling Period**	**PCR Positive Samples/Tested Samples[1] per Sampling Period**	**PCR Positive Samples/Tested Samples[1] per Sampling Period**	**Virus Positive Animals/Tested Animals[2] per Sampling Period**
Prenatal infection (*n* = 27)		≤1	2/7	5/7	n.t.	n.t.	5/9
Prenatal infection (*n* = 27)		4–8	7/7	4/7	2/2	n.t.	3/3
Prenatal infection (*n* = 27)		12–22	7/10	5/7	n.t.	n.t.	n.t.
Prenatal infection (*n* = 27)		35–40	6/10	8/10	1/3	n.t.	7/10
Offspring persistently infected female early embryonic stages (*n* = 44)		unborn	n.t.	n.t.	n.t.	n.t.	44/44
Offspring persistently infected female (*n* = 149)	F1 Generation (*n* = 43)	<1–4	24/25	9/25	5/5	n.t.	27/28
Offspring persistently infected female (*n* = 149)	F1 Generation (*n* = 43)	23–30	4/4	1/4	2/2	4/4	3/3
Offspring persistently infected female (*n* = 149)	F1 Generation (*n* = 43)	32–61	12/12	4/12	11/11	12/12	12/12
Offspring persistently infected female (*n* = 149)	F2 Generation (*n* = 81)	3–10	54/54	0/54	1/1	7/7	10/10
Offspring persistently infected female (*n* = 149)	F2 Generation (*n* = 81)	14–19	8/8	1/7	n.t.	9/9	7/7
Offspring persistently infected female (*n* = 149)	F2 Generation (*n* = 81)	21–28	19/19	1/19	15/15	2/2	19/19
Offspring persistently infected female (*n* = 149)	F3 Generation (*n* = 8)	2–3	8/8	0/8	n.t.	n.t.	8/8
Offspring persistently infected female (*n* = 149)	F4 Generation (*n* = 12)	5–69	10/10	0/8	1/1	3/3	n.t.
Offspring persistently infected female (*n* = 149)	F5 Generation (*n* = 5)	27–49	4/4	0/4	2/2	5/5	n.t.
Offspring persistently infected male[3] (*n* = 92)		<1	2/6	6/6	n.t.	n.t.	2/7
Offspring persistently infected male[3] (*n* = 92)		1	5/39	39/39	n.t.	n.t.	9/66
Offspring persistently infected male[3] (*n* = 92)		2	3/9	9/9	n.t.	n.t.	3/9
Offspring persistently infected male[3] (*n* = 92)		3–4	0/10	10/10	n.t.	n.t.	2/10

[1] For blood and urine, the number of positive samples versus the total number of samples tested during a given time period is shown. Since individuals were sampled up to 3 times per sampling period, the number of tested samples can exceed the number of animals per group. [2] For organs, the number of positive animals versus the total number of tested

animals is shown. The following organs were sampled: liver, spleen, kidney, heart, lung, brain, gonads, salivary glands, eyes, thymus, stomach, prostate, seminal glands, cervical and inguinal lymphnodes, placenta, blastocysts, whole embryos, and fetal livers. Animals were considered positive, if one or more of the tested organs contained infectious virus.
[3] Persistently infected males never had any direct contact to their offspring. The males served as the original source of the virus, and through infecting their female mate, the virus was passed down to the offspring. n.t. = not tested.

Supplementary Table 4. Overview of the neutralization capacity of LASV-specific antibodies.

**Days since first exposure[1]**		**Inoculation[1]**	**Offspring[1]**	**Contact[1]**
<50		*n* = 23	*n* = 3	*n* = 7
	neutralization	0/23	1/3	0/7
	IC_{50} (min – max)	–	1:3	–
50–99		*n* = 4	n.t.	*n* = 7
	neutralization	0/4	n.t.	0/7
	IC_{50} (min – max)	–	–	–
100–200		*n* = 8	*n* = 8	*n* = 1
	neutralization	5/8	1/8	0/1
	IC_{50} (min – max)	1:3 – 1:235	1:128	–
>200		*n* = 2	*n* = 17	n.t.
	neutralization	2/2	2/17	n.t.
	IC_{50} (min – max)	1:19 – 1:255	1:31 – 1:122	–

[1] The time since the first exposure to LASV is depicted in days. Animals were inoculated at various time points (Inoculation), potentially exposed in utero, i.e. borne to persistently infected females or females inoculated during gestation (Offspring), and adults were exposed to infected mates or inoculated offspring (Contact)

Supplementary Table 5. Neutralization capacity of LASV-specific antibodies of animals inoculated with LASV.

Days since inoculation[1]		Age at inoculation (days)[1]	IC50	Viremia status
<50 (*n* = 23)	14	57	–	negative
	14	57	–	negative
	14	57	–	negative
	15	29	–	negative
	15	29	–	negative
	15	29	–	positive
	28	57	–	negative
	28	57	–	negative
	28	57	–	negative
	28	57	–	negative
	29	117	–	negative
	30	29	–	negative
	30	29	–	negative
	30	29	–	negative
	35	29	–	negative
	35	29	–	negative
	35	29	–	negative
	35	57	–	negative
	35	57	–	negative
	35	57	–	negative
	35	57	–	negative
	49	15	–	negative
	49	15	–	positive
50–99 (*n* = 4)	71	11	–	negative
	71	383	–	negative
	71	11	–	positive
	71	11	–	positive
100–200 (*n* = 8)	120	7	–	positive
	127	7	1:235	positive
	158	6	1:32	negative
	158	6	1:3	negative
	158	6	–	negative
	167	11	1:155	positive
	167	11	–	negative
	190	11	1:21	negative
>200 (*n* = 2)	206	7	1:255	positive
	240	7	1:19	positive

[1] The time since the first exposure to LASV is depicted in days. Animals were inoculated s.c., or i.v. (1,000 or 10,000 FFU) with LASV strain Ba366 at either the age of 6–7, 11, 15, 29, 57, 117, or 383 days.

Supplementary Table 6. Neutralization capacity of LASV-specific antibodies of animals borne to infected mothers.

**Days since birth**[1]		**Origin**[1]	**IC50**	**Viremia status**
<50 (*n* = 3)	28	Inoculation of pregnant female	–	positive
	28	Inoculation of pregnant female	1:3	positive
	28	Inoculation of pregnant female	–	positive
100–200 (*n* = 8)	152	Persistently infected mother	1:128	positive
	152	Persistently infected mother	–	positive
	152	Persistently infected mother	–	positive
	155	Persistently infected mother	–	positive
	169	Persistently infected mother	–	positive
	169	Persistently infected mother	–	positive
	174	Persistently infected mother	–	positive
	198	Persistently infected mother	–	positive
>200 (*n* = 17)	245	Inoculation of pregnant female	–	positive
	249	Inoculation of pregnant female	1:122	positive
	251	Inoculation of pregnant female	1:31	positive
	280	Inoculation of pregnant female	–	positive
	280	Inoculation of pregnant female	–	negative
	280	Inoculation of pregnant female	–	negative
	281	Inoculation of pregnant female	–	positive
	281	Inoculation of pregnant female	–	negative
	281	Inoculation of pregnant female	–	negative
	408	Persistently infected mother	–	positive
	422	Persistently infected mother	–	positive
	422	Persistently infected mother	–	positive
	424	Persistently infected mother	–	positive
	424	Persistently infected mother	–	positive
	425	Persistently infected mother	–	positive
	425	Persistently infected mother	–	positive
	425	Persistently infected mother	–	positive

[1] The age at the time of sampling is depicted in days. Animals were borne to either persistently infected mothers, or females that were inoculated during gestation.

Supplementary Table 7. Neutralization capacity of LASV-specific antibodies of adult animals exposed to infected individuals.

Days since first exposure[1]		Origin[1]	IC50	Viremia status
<50 (*n* = 7)	30	Natural exposure	–	negative
	31	Natural exposure	–	negative
	33	Natural exposure	–	negative
	34	Natural exposure	–	negative
	35	Natural exposure	–	negative
	46	Natural exposure	–	negative
	49	Natural exposure	–	negative
50–99 (*n* = 7)	51	Natural exposure	–	negative
	56	Natural exposure	–	negative
	65	Natural exposure	–	negative
	70	Natural exposure	–	negative
	71	Natural exposure	–	negative
	77	Natural exposure	–	negative
	93	Natural exposure	–	negative
100–200 (*n* = 1)	129	Natural exposure	–	negative

[1] Adults were exposed to persistently infected mates or inoculated offspring. Days were counted from the moment of the first exposure, i.e., placement in the same cage or inoculation of offspring.

Supplementary Table 8. Scoring and Humane endpoint criteria.

**Monitoring**	**Score**[1]
**I Bodyweight**<br>compared to starting weight (for adult animals)<br>compared to control group (for neonates/juveniles up to 12 weeks post-birth)	
Unchanged or increase	0
Reduction > 5 %	1
Reduction > 10 %	2
Reduction > 20 %	3
**II General well being**	
Smooth, glossy, close-lying coat; clean orifices	0
dull, matted or ruffled coat; cloudy eyes	1
orifices moist or clotted by secretions; abnormal posture; high muscle tone; dehydration	2
cramps or spasms; paralysis; raspy breathing; hypothermia	3
**III Behaviour**	
normal behaviour (sleep, reaction to stimuli, curiosity, social behaviour)	0
abnormal behaviour, impaired motor skills or hyperkinetics	1
Isolation; signs of pain; apathy; pronounced hyperkinetics or stereotypy; ataxia	2
Automutilation	3
**IV Experiment specific criteria**<br>no experiment specific symptoms are expected	
**Evaluation, measures taken**	**Total Score**
No burden	0
minor burden: continue close monitoring (1x daily),<br>supportive measures (e.g., heat supply, special food, therapy)	1
medium burden: animal is euthanized	2
high burden: not applicable, since animals are euthanized at medium burden	3

[1] Scores (0/1/2/3) are assigned once at least one criteria is fulfilled. If multiple criteria are fulfilled in the same category, only the highest possible score is assigned.

Supplementary Table 9. Overview of animals for which representative IHC and H&E images are shown.

Group	Sex	Age in days	Days post-inoculation	Viremia status[1]	Antibody status[2]	Figures
Uninfected control	Male	72	-	Negative	Negative	Fig 2b; Fig S1 a,b,c; Fig S20
	Female	71	-	Negative	Negative	Fig 2b; Fig S1 b (ovary); Fig S20 (ovary)
Inoculation 1,000 FFU day 6–7	Male	127	120	Positive	Positive	Fig 2; Fig 8 a,b; Fig S1 a,b,c; Fig S19 a,b,c; Fig S21
	Female	247	240	Positive	Positive	Fig 2; Fig 8 b (ovary); Fig S1 b (ovary)
	Female	213	206	Positive	Positive	Fig S1 c (eye); Fig S19 a (eye)
Prenatal infection	Male	245	-	Positive	Negative	Fig 8 a,b; Fig S10; Fig S19 a,b,d,e
	Female	249	-	Positive	Positive	Fig 8 b (ovary); Fig S10 (ovary); Fig S19 a,b
Offspring persistently infected female	Male	425	-	Positive	Negative	Fig 8 b (testis, epididymis, prostate); Fig S16 (testis, epididymis, prostate)
	Female	159	-	Positive	Negative	Fig 8 a,b; Fig S16; Fig S19 a,b,f
	Female	174	-	Positive	Negative	Fig 6 b,c

[1] Viremia status at the time of euthanasia was determined via qPCR. [2] Antibody status (IgG) at the time of euthanasia was determined by ELISA.

Supplementary References

1. Mähler, M. *et al.* FELASA recommendations for the health monitoring of mouse, rat, hamster, guinea pig and rabbit colonies in breeding and experimental units. *Lab Anim* **48**, 178–192 (2014).
