# Supplementary material for: Lassa virus persistence with high viral titers following experimental infection in its natural reservoir host, Mastomys natalensis
Source: Nat Commun. 2024 Oct 29;15:9319. doi: 10.1038/s41467-024-53616-4 (PMC11522386; doi:10.1038/s41467-024-53616-4)
Supplement: Supplementary file 2 — Reporting Summary [file 41467_2024_53616_MOESM2_ESM.pdf]

nature portfolio

Corresponding author(s): Lisa Oestereich

Last updated by author(s): Oct 1, 2024

Reporting Summary

Nature Portfolio wishes to improve the reproducibility of the work that we publish. This form provides structure for consistency and transparency in reporting. For further information on Nature Portfolio policies, see our Editorial Policies and the Editorial Policy Checklist.

Statistics

For all statistical analyses, confirm that the following items are present in the figure legend, table legend, main text, or Methods section.

n/a	Confirmed	
☐	☒	The exact sample size (*n*) for each experimental group/condition, given as a discrete number and unit of measurement
☐	☒	A statement on whether measurements were taken from distinct samples or whether the same sample was measured repeatedly
☐	☒	The statistical test(s) used AND whether they are one- or two-sided<br>*Only common tests should be described solely by name; describe more complex techniques in the Methods section.*
☒	☐	A description of all covariates tested
☐	☒	A description of any assumptions or corrections, such as tests of normality and adjustment for multiple comparisons
☐	☒	A full description of the statistical parameters including central tendency (e.g. means) or other basic estimates (e.g. regression coefficient) AND variation (e.g. standard deviation) or associated estimates of uncertainty (e.g. confidence intervals)
☐	☒	For null hypothesis testing, the test statistic (e.g. *F*, *t*, *r*) with confidence intervals, effect sizes, degrees of freedom and *P* value noted<br>*Give P values as exact values whenever suitable.*
☒	☐	For Bayesian analysis, information on the choice of priors and Markov chain Monte Carlo settings
☒	☐	For hierarchical and complex designs, identification of the appropriate level for tests and full reporting of outcomes
☒	☐	Estimates of effect sizes (e.g. Cohen's *d*, Pearson's *r*), indicating how they were calculated

Our web collection on statistics for biologists contains articles on many of the points above.

Software and code

Policy information about availability of computer code

Data collection	All Data was collected in Microsoft Excel. Counting of spots in the serum neutralisation experiments was done in the AID iSPOT Elispot reader software V7. ELISA results were obtained using the Magellan Software (Tecan). RT-PCR data was analysed using the Rotogene Software (Qiagen).
Data analysis	Data was analysed in Microsoft Excel and Prism 10. Organ heat maps and overview figures were created with Biorender.com.

For manuscripts utilizing custom algorithms or software that are central to the research but not yet described in published literature, software must be made available to editors and reviewers. We strongly encourage code deposition in a community repository (e.g. GitHub). See the Nature Portfolio guidelines for submitting code & software for further information.

Data

Policy information about availability of data

All manuscripts must include a data availability statement. This statement should provide the following information, where applicable:

- Accession codes, unique identifiers, or web links for publicly available datasets
- A description of any restrictions on data availability
- For clinical datasets or third party data, please ensure that the statement adheres to our policy

The data generated in this study are provided in the Supplementary Information and in the Source Data file.

Research involving human participants, their data, or biological material

Policy information about studies with human participants or human data. See also policy information about sex, gender (identity/presentation), and sexual orientation and race, ethnicity and racism.

Reporting on sex and gender	not applicable
Reporting on race, ethnicity, or other socially relevant groupings	not applicable
Population characteristics	not applicable
Recruitment	not applicable
Ethics oversight	not applicable

Note that full information on the approval of the study protocol must also be provided in the manuscript.

Field-specific reporting

Please select the one below that is the best fit for your research. If you are not sure, read the appropriate sections before making your selection.

☒ Life sciences ☐ Behavioural & social sciences ☐ Ecological, evolutionary & environmental sciences

For a reference copy of the document with all sections, see nature.com/documents/nr-reporting-summary-flat.pdf

Life sciences study design

All studies must disclose on these points even when the disclosure is negative.

Sample size	For the inoculation experiments at least two whole litter per experimental were chosen. The number of animals euthanized for terminal sampling varied based on initial litter size and composition, however, for each experimental group at least two individuals were sacrificed per sampling period.
Data exclusions	No data was excluded from the analysis.
Replication	All inoculation experiments were done in at least two independant experiment and with at least two individual per timepoint. All data is shown and the observed variation between the "replicates" is expected. The two independant breeding pairs as a base for each experiments was choosen to reflect the natural variation of the
Randomization	in animal younger than 3 weeks, individuals were choosen at random for sample collection. For animal above 3 weeks, balanced number of male and female (based on litter composition) were sacrificed for each time point.
Blinding	The animal experiments were not blinded as this is not feasible for BSL4 animal experiments.

Reporting for specific materials, systems and methods

We require information from authors about some types of materials, experimental systems and methods used in many studies. Here, indicate whether each material, system or method listed is relevant to your study. If you are not sure if a list item applies to your research, read the appropriate section before selecting a response.

Materials & experimental systems

n/a	Involved in the study	
☐	☒	Antibodies
☐	☒	Eukaryotic cell lines
☒	☐	Palaeontology and archaeology
☐	☒	Animals and other organisms
☒	☐	Clinical data
☒	☐	Dual use research of concern
☒	☐	Plants

Methods

n/a	Involved in the study	
☒	☐	ChIP-seq
☒	☐	Flow cytometry
☒	☐	MRI-based neuroimaging

Antibodies

Antibodies used	Lassa NP antibody L2F1 and 2B5 for virus titration, Lassa GP antibody 22.5D (Ab00225D from Zalgen)
Validation	The L2F1 and 2B5 antibodies have been extensively used in our lab and their specificity has been published. (Hufert 1989). The 22.5D antibody from Zalgen is commercially available and its use for immunohistochemistry has been validated as part if this study (data for controls is shown in Supplementary information)

Eukaryotic cell lines

Policy information about cell lines and Sex and Gender in Research

Cell line source(s)	Vero 81 CRL-1587 cells from ATCC were used for virus amplification and titration.
Authentication	The cells were authenticated by next generation sequencing
Mycoplasma contamination	The cells used in this study were tested negative for mycoplasma.
Commonly misidentified lines (See ICLAC register)	not applicable

Animals and other research organisms

Policy information about studies involving animals; ARRIVE guidelines recommended for reporting animal research, and Sex and Gender in Research

Laboratory animals	The study was performed with Mastomys natalensis breed at BNITM. Age of experimental animals ranged from 1 day to 425 days. The age is indicated in the figures, text and detrailed in the appendix.
Wild animals	not applicable
Reporting on sex	In general both sex were considered and (if possible) all groups had a balanced number of male/females. Sex specific data is shown (e.g. virus titers in reproductive organs) and the information of the sex of the animal e.g. for the histology data is given as an supplementary table.
Field-collected samples	not applicable
Ethics oversight	The study was carried out in strict compliance with the recommendations of the German Society for Laboratory Animal Science under the supervision of a veterinarian. All protocols were approved by the Committee on the Ethics of Animal Experiments of the City of Hamburg (N 028/2018, N 050/2021, N 051/2021 and O42/2018).

Note that full information on the approval of the study protocol must also be provided in the manuscript.

Plants

Seed stocks	not applicable
Novel plant genotypes	not applicable
Authentication	not applicable
